# Exploring the Dynamic Coordination Sphere of Lanthanide Aqua Ions: Insights from r^2^SCAN-3c Composite-DFT Born–Oppenheimer Molecular Dynamics Studies

**DOI:** 10.1021/acsomega.4c04947

**Published:** 2024-12-16

**Authors:** Emiliano Isaías Alanís-Manzano, C. I. León-Pimentel, Laurent Maron, Alejandro Ramírez-Solís, Humberto Saint-Martin

**Affiliations:** † Instituto de Ciencias Físicas, Universidad Nacional Autónoma de México, Cuernavaca, Morelos 62210, México; ‡ Departamento de Matemáticas/Fisicoquímica, Facultad de Química, Universidad Nacional Autónoma de México, Ciudad de Mexico 04510, México; § INSA Laboratoire de Physicochimie de Nano-Objets, Université de Toulouse, 135 Avenue de Rangueil, F31077 Toulouse, France; ∥ Depto. de Física, Centro de Investigación en Ciencias-IICBA Universidad Autónoma del Estado de Morelos, Cuernavaca, Morelos 62209, México

## Abstract

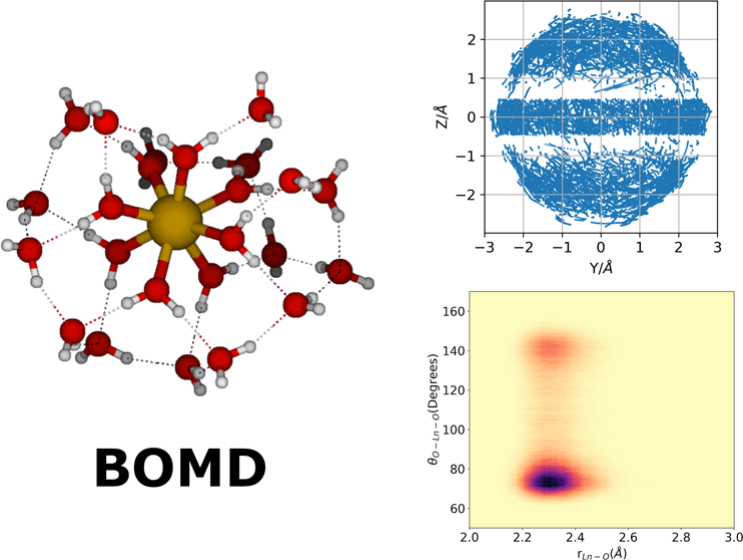

Born–Oppenheimer molecular dynamics (BOMD) simulations were performed to investigate the structure and dynamics of the first hydration shells of five trivalent lanthanide ions (Ln^3+^) at room temperature. These ions are relevant in various environments, including the bulk aqueous solution. Despite numerous studies, accurately classifying the molecular geometry of the first hydration sphere remains a challenge. To addres this, a cluster microsolvation approach was employed to study the interaction of Ln^3+^ ions (La, Nd, Gd, Er, and Lu) with up to 27 explicit water molecules. Electronic structure calculations were performed with the composite r^2^SCAN-3c method. The results demonstrate that this method offers an optimal balance between precision and computational efficiency. Specifically, it accurately predicts average Ln–O distances (MAE = 0.02 Å) of the first hydration sphere and preferred coordination numbers (CN) for the different lanthanide cations as compared to reported data in bulk. Highly dynamic first hydration shells for the examined Ln^3+^ ions were found, with noticeable and rapid rearrangements in their coordination geometries, some of which can be recognized as the tricapped trigonal prism (TTP) and the capped square antiprism (CSAP) for CN = 9, and as the square antiprism (SAP), the bicapped trigonal prism (BTP), and the trigonal dodecahedron (DDH) for CN = 8. However, ca. 70% of the nonacoordinated configurations did not meet the criteria of TTP or CSAP structures. For CN = 8, the percentage of configurations that could not be assigned to SAP, BTP, or DDH was lower, around 30%. The theoretical EXAFS spectra obtained from the BOMD simulations are in good agreement with the experimental data and confirm that model microsolvated environments accurately represent the near-solvation structure of these trivalent rare-earth ions. Moreover, this demonstrates that the faster dynamics of the first hydration shell can be studied separately from the dynamics of water exchange in the bulk aqueous solution.

## Introduction

The identification of lanthanide coordination structures and dynamics at atomic resolution holds immense significance across a wide range of technological applications.^1^ These applications encompass electric car batteries, single-molecule magnets, medical contrast agents, high-energy-density capacitors, rare-earth separation and purification, luminescence, and various other technological domains.^2−7^ Lanthanide ions in solution exist predominantly in the + III oxidation state, establishing complexes with both ligand and solvent molecules. Ln cations exhibit characteristics of reactive Lewis acids, often displaying greater reactivity compared to certain 3d transition metals.^8^ In solution, they mainly participate in the formation of labile ionic coordination bonds, with coordination numbers typically ranging from 3 to 12.^9^ This gives rise to notably reactive and dynamic coordination structures that span a rather large conformational space. Consequently, deciphering the coordination structure of Ln^3+^ -ligand complexes in solution presents a formidable challenge, both from the experimental and computational perspectives.^1,10^ In this study, we focus on the hydration structure and dynamics of lanthanide aqua ions at room temperature.

Extended X-ray absorption fine structure (EXAFS) spectroscopy,^11^ neutron diffraction,^12^ and Raman spectroscopy^13,14^ data in solution and reliable computational models have been used to obtain accurate Ln–O distances and reliable coordination numbers (CN) for lanthanide aqua ions. Early lanthanides (La–Nd) exhibit a CN = 9, while late lanthanides (Tb–Lu) show a CN = 8. Intriguingly, the middle lanthanides exhibit a dynamic equilibrium between 9-fold and 8-fold coordination structures, leading to an average noninteger CN.^9^

Despite extensive knowledge of water coordination numbers, accurately classifying the molecular geometry of the first hydration sphere of Ln^3+^ ions remains a significant challenge.^10,15,16^ Identifying the configuration of a multiply charged metal-aqua ion in solution is particularly difficult due to the inherent thermal structural disorder in the liquid state and, sometimes, from the detachment of water molecules from the aqua ion.^15^ These factors are important and lead to a not uniquely defined coordination geometry of the Ln^3+^ ion in solution, thereby hindering its accurate identification. This challenge arises in stark contrast to the simple models drawn from the arrangement of water molecules around singly or even doubly charged cation. Additionally, there is recent evidence showing that hydrated Ln^3+^ species might not possess a defined molecular geometry but are rather fluxional.^10^ Thus, the labile quality of Ln–O coordination bonds, coupled with the presumably dynamic nature of the first hydration shell, renders the clarification of the molecular geometry of this shell a profoundly challenging endeavor.

From the theoretical perspective, mostly molecular dynamics (MD) simulations using classical, ab initio, or mixed quantum/classical force fields (FF) have been used to investigate the hydration structure and dynamics of lanthanide aqua ions. Classical MD simulations can replicate the experimental Ln-O distances and the CN along the series. However, it is important to note that the configuration of the hydration polyhedron in the first coordination sphere of Ln(III) ions has been extensively debated in the literature.^10,15,17^ Most studies, both classical molecular dynamics and experimental, conclude that light lanthanides exhibit a nonacoordinated trigonal tricapped prism (TTP) geometry, while heavy lanthanides adopt an octacoordinated square antiprism (SAP) geometry.^17−22^ Classical MD simulations offer the advantage of accessing larger time scales, thus, enabling the capture of water exchange and ion association events in aqueous solvated systems. However, a crucial matter emerges as classical MD force fields rely on parameter fitting, which may not accurately represent the lanthanide coordination properties in chemical environments distinct from those used during a particular parametrization scheme. Furthermore, these force fields do not account for reactivity due to the absence of explicit electronic structure treatment. In this study, we aim to use state-of-the-art computational quantum chemistry in order to shed some light on the dynamic coordination structure of the lanthanide aqua cations.

Recent ab initio molecular dynamics (AIMD) simulations combined with EXAFS experimental results conclude that all Ln^3+^ ions have highly dynamic first coordination spheres.^10^ In essence, solvated Ln^3+^ species lack a fixed molecular geometry and instead exhibit fluxional character. Additionally, they demonstrate the utility of EXAFS spectroscopy in characterizing the coordination structure of these ions in solution, providing valuable information about their behavior in aqueous environments.

For the reasons mentioned above, this work has two goals: (a) to demonstrate that the new composite r^2^SCAN-3c method coupled with Born–Oppenheimer molecular dynamics (BOMD) simulations can accurately describe the coordination properties of Ln^3+^ ions in aqueous solutions; (b) to resolve the dynamical structure at room temperature of these ions through BOMD simulations. Here we present a detailed study of the structural and dynamic properties of Ln(H_2_O)_27_^3+^ (Ln = La, Nd and Gd) and Ln(H_2_O)_24_^3+^ (Ln = Er and Lu) clusters through BOMD simulations.

Although it is well known that ion solvation in electrolyte solutions can be significantly influenced by long-range interactions, our previous BOMD simulations have demonstrated that they accurately represent cations with fully formed first hydration spheres, free of ion pairs.^23−31^ While long-range interactions, such as those involving distant water molecules or weakly coordinated counterions,^32^ may influence ion distribution in solution, there are cases—particularly in biochemical environments—where ion coordination is strongly influenced by the first shell of ligands. The binding conformation of ligands and their atomic speciation within this first sphere are critical, as ligands may rearrange upon binding to the Ln ion.

In such situations, the binding conformation of the primary hydration shell can be crucial for distinguishing ions that might be chemically similar, particularly in the selectivity of certain ion channels.^33−35^ Additionally, the structure of both the first and second coordination spheres must be considered, as they influence each other.^1,36^ This interplay is especially relevant in Ln^3+^-based enzymatic catalysis in some methylotrophic bacteria,^7^ where variations in coordination can affect catalytic efficiency. Therefore, it is crucial to characterize coordination differences as ions transition from bulk aqueous solutions to water clusters, as seen in our previous studies on Pb^2+^.^23,25^ Our BOMD cluster approach has proven reliable for studying nanosolvation in various metallic systems.^23−31^ This work focuses on the first hydration shell of Ln^3+^ cations influenced by the surrounding water molecules at room temperature.

## Computational Details

Geometry optimizations and frequency computations were performed with the ORCA-5 program package^37−39^ (version 5.0.3). Fully unrestricted quantum-chemical geometry optimizations of Ln(H_2_O)_9_(H_2_O)_18_^3+^ (Ln = La or Nd), Gd(H_2_O)_8_(H_2_O)_19_^3+^, and Ln(H_2_O)_8_(H_2_O)_16_^3+^ (Ln = Er or Lu) were carried out using Kohn–Sham density functional theory (DFT) with two different hybrid exchange-correlation (XC) functionals (B3LYP^40^ and TPSS^41^), in conjunction with the aug-cc-pVDZ^42,43^ basis sets for the oxygen and hydrogen atoms. Additionally, the D3-BJ empirical dispersion correction by Grimme^44^ with Becke–Johnson damping was applied. It is worth noting that the B3LYP^36,45−47^ and TPSS^36^ XC functionals have previously proven successful in static studies of structural and thermodynamic properties of Ln^3+^ aqua ions. We also explored the performance of the new electronic structure r^2^SCAN-3c composite method.^48^ In this scheme the r^2^SCAN meta-generalized-gradient approximation^49^ (mGGA) functional is combined with a tailor-made triple-ζ Gaussian atomic orbital basis set^48^ as well as with refitted D4^50−52^ and geometrical counterpoise^53^ corrections for London-dispersion and basis set superposition error (BSSE). The Stuttgart-Köln relativistic effective core potentials (RECP) and their associated basis sets for the Ln^3+^ ions were employed. For DFT/aug-cc-pVDZ (DFT = B3LYP or TPSS), the large-core 4f in-core RECP was utilized^54,55^ with the associated (8s7p6d3f2g)/[6s5p5d3f2g] ECPxxMWB-II basis set,^54,56,57^ where xx = 46 + *n*, and *n* represents the number of 4f electrons in the ground state electronic configuration of the trivalent lanthanides, i.e., [Xe]4*f*^*n*^ (*n* = 0–14). The r^2^SCAN-3c composite method uses the large-core ECP46MWB for La^3+^ and the small-core ECP28MWB^58−60^ for Ce^3+^-Lu^3+^ along with the def2-mTZVPP basis set.^48^ r^2^SCAN-3c computations were accelerated using the resolution of the identity (RI-J) approximation,^61^ with the def2-mTZVPP/J^48^ auxiliary basis set for all atoms in the system.

As shown in the Supporting Information, the composite method r^2^ SCAN-3c has been shown to accurately yield bond distances of the lanthanide hydration environment (Supporting Information, Tables S2–S5). Comparisons with DFT data as well as experimental Ln–O distances validate its accuracy. In particular, the Mean Absolute Error (MAE) of the predicted Ln-O distances is 0.02 Å.

The Born–Oppenheimer molecular dynamics (BOMD) simulations were carried out with the ORCA-5 molecular dynamics module.^37^ We employed a time step of 0.5 fs with the velocity-Verlet algorithm as the time integration method. The temperature was controlled at *T* = 300 K by a chain of four Nosé–Hoover thermostats with a high-order Yoshida integrator and a 10 fs coupling constant. After an assessment of the Ln–O distances and hydration free energies predicted by the different levels of theory explored in this work, the composite r^2^SCAN method was chosen to perform the BOMD simulations. All of the Ln(III) microsolvated ions were simulated for at least 19 ps after a thermalization period of 1 ps. Though our BOMD simulations do not sample large enough time scales to capture water exchange events, they combine the advantage of taking into account the electronic structure with molecular dynamics sampling of the system. This combined approach is needed to capture the expected fluxional character of the first hydration sphere. As is customary for simulations of the gas phase, the system was coupled only to a thermostat, not to any barostat, and because no boundaries were imposed, the volume was considered to be large enough to allow for free expansion so that the molecules included are held together solely by their interactions. Of course, it could be argued that the nanodroplet will eventually evaporate and the molecules will drift apart to a maximum entropy state. Nonetheless, the time scale in which evaporation occurs is long enough to allow for both theoretical and experimental studies of small molecular clusters, especially for phenomena with characteristic time scales of tens of picoseconds.^62^

### Configurational Search of Low-Energy Ln(H_2_O)_*k*_^3+^ Clusters (*k* = 24 and 27)

Though the BOMD with the Nosé–Hoover thermostat produces a valid sample of the NPT ensemble, convergence to a set of statistically meaningful configurations requires starting from low-energy structures for our microsolvated Ln(H_2_O)_*k*_^3+^ model. Although Kuta and Clark present optimal structures in vacuum with two explicit hydration spheres,^36^ their configurational search was by no means exhaustive. The global optimization of molecular aggregates of that size becomes a nontrivial task because their potential energy surface is very flat and has a large number of possible low-energy isomers.

To generate candidates for global minima using two explicit hydration spheres, we started from the octacoordinated and nonacoordinated optimal structures for La^3+^, Gd^3+^, and Lu^3+^ previously reported.^63,64^ These structures were kindly provided by Michael Dolg, and the MP2-optimized structures are shown in Figure S1.

Starting from these molecular geometries, we performed the following procedure, described here for nonacoordinated Lanthanum:

1. The optimal structure in vacuum was surrounded by 18 water molecules, distributed arbitrarily within a sphere of radius 4 Å. The ABCluster program was used to generate 10–20 structures of this kind.^64,65^
2. The generated structures were visually inspected to eliminate atypical ones, and 10 potential candidates are selected. For instance, an atypical structure could be one with excessively large Ln–O distances.
3. We partially optimized the geometry of these 10 candidates at the r^2^SCAN-3c level, employing loose convergence criteria.
4. Subsequently, we identified between two and three aggregates with the lowest energy and conducted full geometry optimizations using r^2^SCAN-3c.
5. The cluster with the lowest energy was confirmed to be a true minimum through vibrational analysis to ascertain the character of the stationary point.
6. This lowest-energy minimum served as the initial configuration of the BOMD.

**Figure 1:**
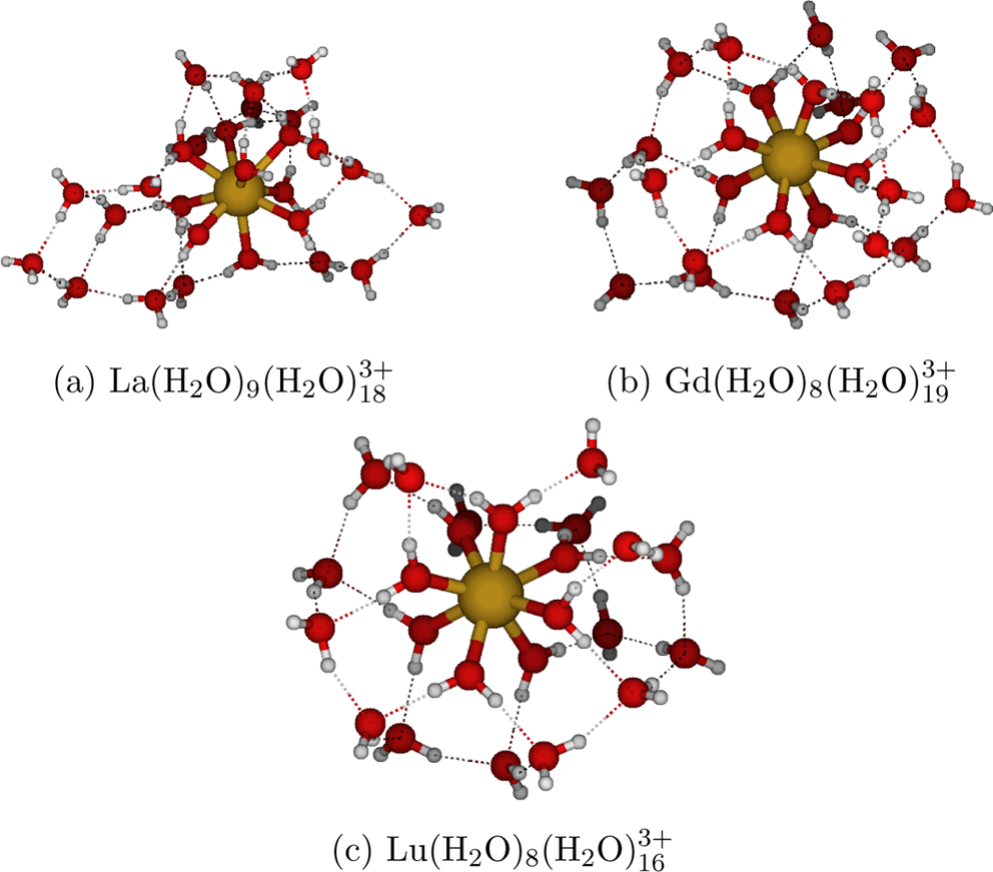
Lowest-energy isomers of the microsolvated complexes of a) La^3+^, b) Gd^3+^ and c) Lu^3+^, obtained at the r^2^SCAN-3c level of theory through our conformational search procedure. These configurations served as the initial structures for the BOMD simulations.

This procedure was carried out for nonacoordinated La and octacoordinated Gd and Lu trivalent ions (see Figure S1), varying both the number of water molecules and the initial solvation sphere radius to match the respective coordination number and average experimental distance in each case.^66^ Therefore, three optimal microsolvation structures for La(H_2_O)_9_(H_2_O)_18_^3+^, Gd(H_2_O)_8_(H_2_O)_19_^3+^ and Lu(H_2_O)_8_(H_2_O)_16_^3+^ were obtained (Figure 1). La(H_2_O)_9_(H_2_O)_18_^3+^ structure is shown in Figure 1a, Gd(H_2_O)_8_(H_2_O)_19_^3+^ in Figure 1b, and Lu(H_2_O)_8_(H_2_O)_16_^3+^ in Figure 1c.

Due to the computational cost of the above configurational search, all further simulations started from an initial configuration produced by the approach proposed by Shiery et al.^1,10^ In order to generate initial configurations for Nd(H_2_O)_9_(H_2_O)_18_^3+^ and Er(H_2_O)_8_(H_2_O)_16_^3+^, we substituted La^3+^ and Lu^3+^ ions in the lowest-energy isomers La(H_2_O)_9_(H_2_O)_18_^3+^ and Lu(H_2_O)_8_(H_2_O)_16_^3+^ with Nd^3+^ and Er^3+^ ions, respectively. We then followed the protocol proposed by Shiery et al. adapted to our BOMD simulations: (I) Initial geometry optimization at 0 K. (II) BOMD simulation for 1 ps with a time step of 1 fs at 500 K. (III) Slow annealing to 0 K over 2 ps using a time step of 1 fs. (IV) Final geometry optimization. The resulting geometry was then used as the starting configuration for the BOMD simulations at *T* = 300 K.

As mentioned at the end of the Introduction, this work focuses on the structure and dynamics of the first hydration shell at room temperature considering the interactions with the water molecules that are in the second hydration shell in the initial configuration. This choice is justified by the fact that the first hydration shell remains isolated from the rest for tens of ps in simulations of bulk aqueous solution, as can be deduced from the Ln^3+^–O radial distribution functions^10,32,67,68^ and from the reported residence times.^69^

### Reference Polyhedra

The Ln-O average distances were used to generate reference molecular geometries for each Ln ion with the aid of the AFICS (Analysis of the First Ion Coordination Sphere) tool of Cantu et al.^70^ The reference molecular geometries of interest here are capped square antiprism (CSAP) and tricapped trigonal prism (TTP) for nonacoordinated lanthanides, while for octacoordinated lanthanides we have square antiprism (SAP), bicapped trigonal prism (BTP) and trigonal dodecahedron (DDH) structures (see Figure S12). In the case of TTP, CSAP, and BTP polyhedra, two characteristic Ln^3+^–O distances are observed. As initially proposed by Cantu et al.,^70^ the optimal reference geometries for these polyhedra are designed to yield the best fit of the average of all ion-oxygen distances with the first *g*(*r*) peak of the corresponding radial distribution function (RDF) resulting from the simulation. Here we adapted their code^70^ to generate our reference TTP, CSAP, and BTP polyhedra. This adaptation ensured a specific  ratio of 1.04, consistent with both experimental neutron diffraction results^69^ and previous molecular dynamics simulations.^71^ We present a comparison of the theoretical Combined Distribution Funtions (CDFs) for Cantu’s TTP, CSAP, and BTP polyhedra with those for our TTP, CSAP, and BTP polyhedra in the Supporting Information (Figures S13 and S14). The oxygen coordinates from the reference molecular geometries were used to calculate the root-mean-square deviations (RMSD) following the procedure utilized by Shiery et al.^10^ but incorporating the modified distances as explained above. The coordinates for the modified Nd^3+^ and Er^3+^ reference molecular geometries are provided in the Supporting Information. The SAP was not modified, as it possesses only one characteristic distance. We calculated the RMSD between each BOMD frame and all reference molecular geometries considered in this study (Supporting Information, Figures S16–S20). These values were averaged over a BOMD trajectory of at least 10 ps, aiming to quantify the similarity of the first solvation shell of each lanthanide ion to a specific reference molecular geometry (Supporting Information, Table S6).

### Internal Dynamics of the First Hydration Shell

To investigate the internal dynamics of the first coordination sphere during the BOMD simulations, we employed a method outlined by Tani and Floris.^72^ For a comprehensive understanding, we refer the reader to the original publication. In essence, this approach involves diagonalizing the inertia tensor of the polyhedron created by the oxygen atoms in the first hydration shell. Comparing the resulting eigenvalues, we identify the axis that stands apart from the others. The eigenvector corresponding to this eigenvalue becomes the *z*-axis of a reference frame centered on the polyhedron’s center of mass. While Tani and Floris exclusively address CN = 9 cases, their methodology is easily applicable to CN = 8 cases as well. Using the chosen eigenvector, which can be referred to as the main inertial axis, along with the other two eigenvectors, we calculate the similarity index (SI) defined as follows:
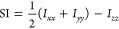
1Here, *I*_*xx*_, *I*_*yy*_, and *I*_*zz*_ represent the respective eigenvalues of the inertia tensor.

For a more detailed structural classification, for each CN, we categorize structures based on their count of oxygen atoms in the equatorial region (within a cutoff of ±10° from the plane orthogonal to the selected axis and encompassing the center of mass). As noted by Tani and Floris, this cutoff effectively excludes prismatic oxygen atoms from the count. When CN = 9, we designate configurations with three equatorial oxygen atoms as TTP-like and those with zero atoms as CSAP-like. Conversely, for CN = 8, we identify structures containing either two or zero equatorial oxygen atoms. In this scenario, we label the former as BTP-like and the latter as SAP-like. This methodology enables us to distinctly categorize two unique structures for each CN value. In-house Python scripts (available upon request from the authors) were employed for the preparation, automation, and analysis stages of the process. To identify the molecular structure of the trigonal dodecahedron (DDH), an additional geometrical analysis was conducted along frames that do not resemble CSAP- or BTP-like structures.

### Theoretical EXAFS Spectra

The calculation of theoretical EXAFS spectra was done according to the procedure proposed by Merkling et al.^73^ The same technique was successfully applied in the study of the solvation of several metallic ions and molecules.^24−27,74,75^ The average EXAFS spectrum for each ion is calculated as the average of spectra obtained from 500 completely decorrelated configurations sampled in the BOMD simulations after the thermalization period. The parameters used for the calculation of the EXAFS spectra (path length, amplitude reduction factor, etc.) were the same as the ones employed by Shiery et al.^10^ The calculations were performed with the FEFF85L code.^76^ For comparison purposes, the experimental EXAFS spectra were taken from refs (10,19,77) employing the WebPlotDigitizer^78^ software.

## Results and Discussion

**Figure 2:**
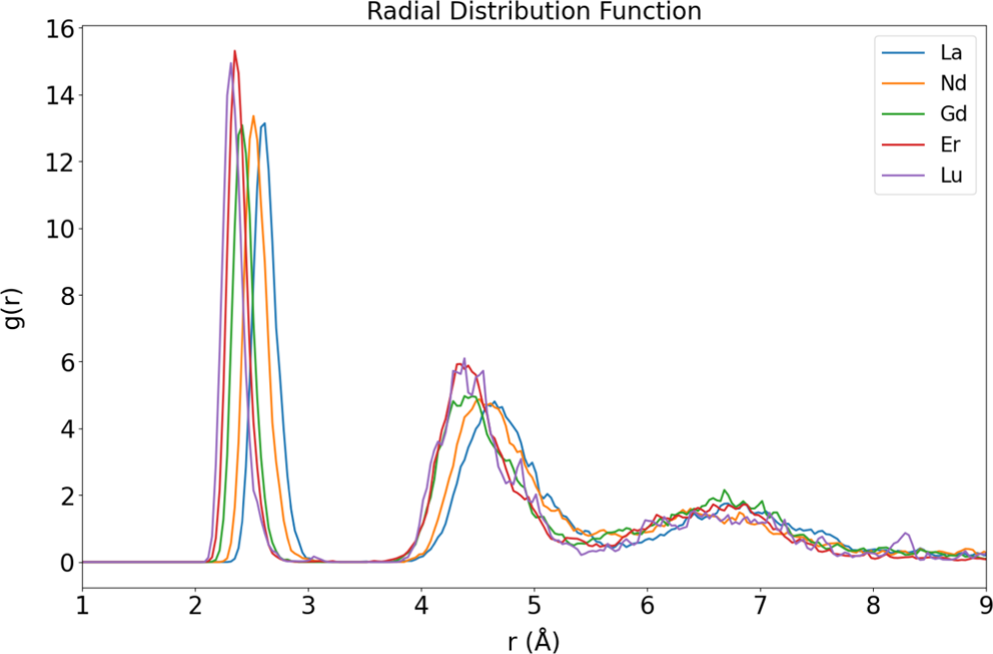
BOMD Ln-O radial distribution functions for the Ln^3+^ ions studied here.

A minimum of 19 ps of stable production BOMD simulations were conducted to analyze the dynamics of Ln^3+^ aqua ions (Supporting Information, Figures S2–S6). Our initial analysis involved computing the *g*_Ln–O_(*r*) radial distribution functions (RDFs) depicted in Figure 2. The distance corresponding to the first peak of each RDF (Supporting Information, Figures S7–S11) is compared to the experimental data in Table 1; the respective coordination numbers (CN) were determined by integrating each curve over the relevant range of *g*_Ln–O_(*r*), from zero to the first minimum for the first coordination shell and from the second minimum to the third minimum for the second coordination shell.

**Table 1:** Structural Parameters Extracted from the RDFs of the Microsolvated Ln^3+^

	first shell		second shell	
	⟨*r*_Ln–O_⟩ (Å)	N_water_	⟨*r*_Ln–O_⟩ (Å)	N_water_
La^3+^				
	2.62	9.0	4.65	10.36
exp	2.56^a^	9.13^b^	4.65^c^	18^c^
Nd^3+^				
	2.52	9.0	4.52	11.28
exp	2.51^a^	8.9^b^	4.60^c^	18^c^
Gd^3+^				
	2.42	8.0	4.45	10.30
exp	2.43^a^	8.7^b^	4.53^c^	18^c^
Er^3+^				
	2.35	8.0	4.35	10.11
exp	2.37^a^	8.19^b^	4.49^c^	14–16^c^
Lu^3+^				
	2.32	8.0	4.38	9.83
exp	2.33^a^	7.97^b^	4.45^c^	14–16^c^

a Experimental average values of all of the Ln–O EXAFS distances and the XRD Ln–O distances reported in the literature.^19,79−84^

b XRD experimental data.^79−81^

c Estimated values by Smirov and Trostin.^66^

The first hydration shell Ln–O distances predicted from the RDFs of the BOMD simulations show very good agreement with the experimental values. The experimental ⟨*r*_Ln–O_⟩ value of the first hydration shell presented in Table 1 represents the average of all of the Ln–O EXAFS distances and the XRD Ln–O distances reported in the literature.^19,79−84^ The highest error, 0.06 Å, is observed for La^3+^, while Nd^3+^, Gd^3+^, and Lu^3+^ exhibit smaller errors of 0.02 or 0.01 Å. This discrepancy arises from the use of a large-core ECP for La^3+^, whereas the small-core ECP is employed for Ce^3+^–Lu^3+^ in the r^2^SCAN-3c method. This situation, where large-core ECP tends to exaggerate the Ln–O distances, is thoroughly documented in static DFT studies concerning water solvation for Ln^3+^.^85^

As in our previous microsolvation studies of metal ions, successfully reproducing the locations and widths of the first and second maxima of the *g*_LnO_(*r*) reported for bulk aqueous solution,^10,32,67,68^ supports our hypothesis that the features of the first hydration shell can be reliably described with the BOMD simulations of the small cluster model at the r^2^SCAN-3c level. This validates again the nanodroplet approach to address the finite temperature structure of the microsolvated Ln^3+^ ions. Nevertheless, third peaks appear that integrate to approximately 8 for La^3+^ and Gd^3+^, around 7 for Nd^3+^, and around 6 for Er^3+^ and Lu^3+^, implying that 6–8 water molecules migrate away from the second shell but lead to a third solvation shell for the simulated time. This phenomenon does not occur in the bulk aqueous solution,^10,32,67^ where the value *g*_LnO_(*r*) = 1 is attained at *r* ≈ 6 Å. One plausible explanation, compatible with the configurations depicted in Figure 1, is that while the water molecules in the first shell are held by the ion-dipole interaction, with significant water–water repulsion, those in the second shell are subject to a more complex combination of attractive ion-dipole and hydrogen-bond interactions with some dipole–dipole repulsion. At distances of >5.5 Å, hydrogen bonding is the main interaction.

At first glance, the number of water molecules in this study might seem too low, as one might argue that having only 16–18 water molecules beyond the first hydration shell is insufficient for accurately modeling the near hydration structure. To address this concern, additional simulations were performed, demonstrating that within tens of picoseconds, the structure and dynamics of the first hydration shell of these highly charged cations are influenced solely by local interactions between the cation and the water molecules in the first and second shells. These simulations confirm that the second shell accommodates the experimentally determined number of water molecules. Detailed results are provided in the SI (Figures S21–S23 and Tables S7 and S8). The key finding is that the structure and dynamics of the first hydration shell remain unchanged

### Structure of the First Hydration Shell

#### Combined Distribution Functions

D’Angelo et al. have proposed a procedure to identify the coordination geometry of a metal ion in a simple and unambiguous manner.^16^ This procedure is based on obtaining Combined Distribution Functions (CDFs) from molecular dynamics simulations, which are two-dimensional probability functions that combine ion-ligand distances and ligand-ion-ligand angles. These CDFs have been effectively used to determine the reference polyhedra of the first hydration layer for La(III), Lu(III) ions,^16^ and other metals that present elusive aqueous coordination structures.^16,86^ Therefore, we present the CDFs of the lanthanide aqua cations from our BOMD simulations.

**Figure 3:**
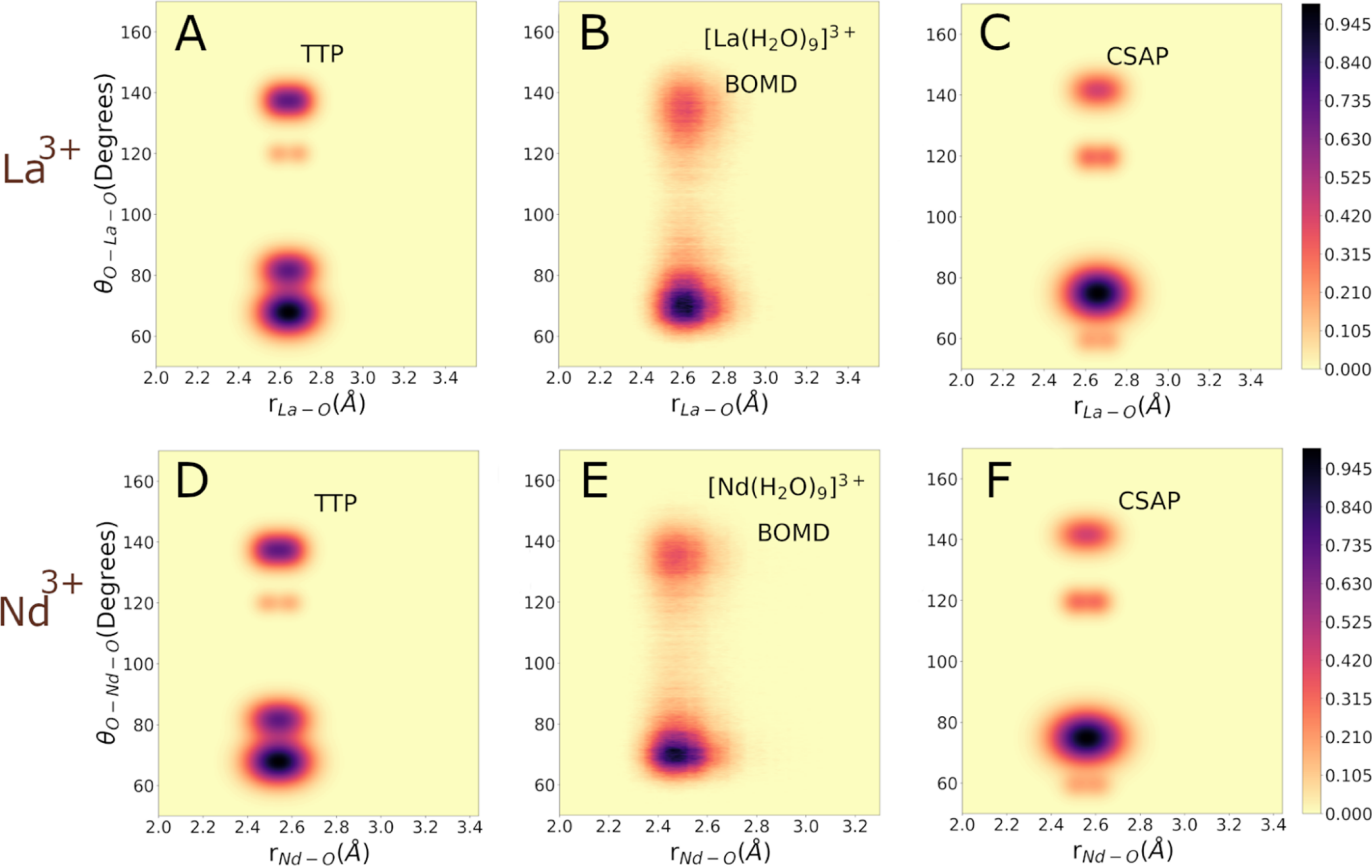
Combined distribution functions (CDFs) for Ln(H_2_O)_9_^3+^ complexes with La^3+^ or Nd^3+^ Ions. Panels (A) and (D): Theoretical CDFs was evaluated for a TTP model of the Ln(H_2_O)_9_^3+^ complexes, where Ln represents La or Nd. Panels (B) and (E): CDFs between the Ln–O distances and the O–Ln–O angles computed from the BOMD simulations of the microsolvated La^3+^ and Nd^3+^ ions. Only oxygen atoms of the first hydration shell are included in the evaluation. Panels (C) and (F): Theoretical CDFs evaluated for a CSAP model of the Ln(H_2_O)_9_^3+^ complexes, where Ln represents La or Nd.

**Figure 4:**
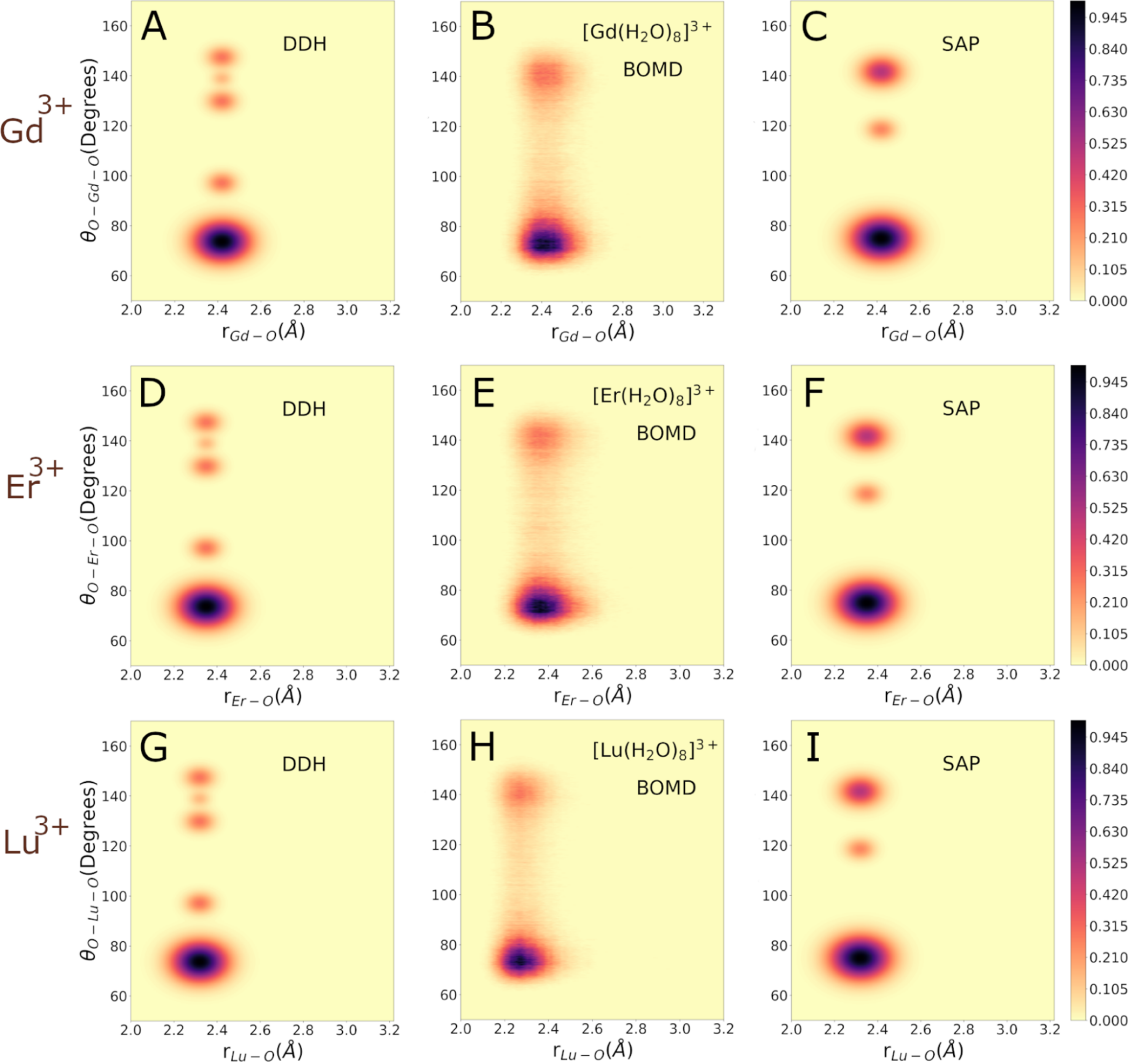
Combined distribution functions (CDFs) for Ln(H_2_O)_8_^3+^ complexes with Gd^3+^, Er^3+^ or Lu^3+^ Ions. Panels (A), (D), and (G): Theoretical CDFs for a DDH model of the Ln(H_2_O)_9_^3+^ complexes, where Ln represents Gd, Er, or Lu. Panels (B), (E), and (H): CDFs between Ln–O distances and O–Ln–O angles computed from the BOMD simulation of the microsolvated Gd^3+^, Er^3+^, or Lu^3+^ ions. Only oxygen atoms of the first hydration shell are included in the determination of the CDFs. Panels (C), (F), and (I): Theoretical CDFs for a SAP model of the Ln(H_2_O)_9_^3+^ complexes, where Ln represents Gd, Er, or Lu.

Based on the work of D’Angelo,^16^ the procedure to generate the CDF was implemented in Python. These CDFs offer precise insights into the coordination geometry of the first solvation shell, allowing us to differentiate between similar structures.^16^ In order to illustrate this, our CDF results are shown in Figures 3 and 4 for CN = 9 and 8, respectively. For CN = 9 a characteristic L-shaped peak is observed at lower angles, in agreement with previous classical MD simulations involving the 9-fold La^3+^^16^ and Ce^3+^^87^ cases. This CDF pattern implies a TTP structure of the first hydration shell. Nevertheless, the characteristic picture of an ideal CSAP is also reflected in both La^3+^ and Nd^3+^ CDFs. This represents a case where at least two molecular geometries undergo noticeable and rapid rearrangements in aqueous environments. It should be noted that even an ideal TTP can be considered approximately as a 3-fold degenerate CSAP, where each of the three capping ligands of the TTP can form the capping ligand of a CSAP.^71^ On the other hand for CN = 8 (Figure 4), a strikingly similar angular distribution emerges. This contradicts prior investigations concerning the aqueous solvation of the Lu^3+^ ion, for which only three prominent peaks on the CDF have been identified, consistent with the SAP symmetry of water molecules. Thus, for CN = 8, the molecular geometries exhibit noticeable and rapid rearrangements, populating at least three reference configurations (SAP, BTP, and DDH). A characteristic theoretical CDF for the BTP geometry is provided in the Supporting Information (Figure S14). In essence, our CDFs for the BOMD simulations shows a rather dynamical character of the angular distribution that precludes the assignment of a single reference molecular geometry.

### Internal Dynamics of the First Hydration Shell

The results of the classification based on the diagonalization of the inertia tensor for the polyhedron formed by the oxygens of waters belonging to the first hydration shell are presented in Tables 2 and 3 for CN = 9 and 8, respectively. We emphasize that when CN is equal to 8, it is also possible to have a trigonal dodecahedron (DDH) molecular structure. Additionally, when we apply the procedure mentioned above to an ideal DDH, it becomes apparent that this type of structure contains zero equatorial oxygen atoms, similar to the SAP configuration. We conducted additional geometrical analysis to classify structures resembling the trigonal dodecahedron (DDH) along frames distinct from CSAP- or BTP-like configurations. The identification of DDH-like structures was based on the geometrical criterion of two perpendicular planes, each containing four oxygen atoms and both comprising the lanthanide.^88^

**Table 2:** Relative Frequencies of Structures Presented as Percentages (%) and Categorized Based on Negative and Positive Similarity Index (SI) Values, as well as TTP-like and CSAP-like Structures^a^

	SI < 0	SI > 0	TTP	CSAP	CSAP_ax_	TTP(SI < 0)	CSAP(SI > 0)
La	54.2	45.8	17.7	14.0	21.6	39.8	31.4
Nd	52.2	47.8	18.8	12.8	20.2	45.0	30.0

a For CSAP-like structures, the percentage (%) of cases in which the capped oxygen is situated within a 10° cone around the main inertial axis (CSAP_ax_) is also provided. The last two columns display the relative frequencies (%) of TTP-like structures with SI < 0 and CSAP-like structures with SI > 0.

**Table 3:** Relative Frequencies of Structures Are Presented as Percentages (%) and Categorized Based on Negative and Positive Similarity Index (SI) Values, as well as BTP-like and SAP-like Structures^a^

	SI < 0	SI > 0	BTP	SAP	BTP (SI < 0)	SAP (SI > 0)	DDH
Gd	68.5	31.5	24.9	34.9	92.4	66.4	7.7
Er	60.2	39.8	21.9	43.1	88.5	73.1	8.3
Lu	66.2	33.8	25.3	39.6	90.4	65.4	8.0

a The two following columns display the relative frequencies (%) of BTP-like structures with SI < 0 and SAP-like structures with SI > 0. The last column has the relative frequencies of putative DDH-like structures found by applying the geometrical criterion of two perpendicular planes, each with four oxygens and both comprising the lanthanoid.^88^

Both La^3+^ and Nd^3+^ ions exhibit an occurrence of approximately 18% TTP-like geometries during the BOMD, while they show an occurrence of only 13% for the CSAP-like geometries. Now, for the octacoordinated ions, the relative frequency of SAP-like structures is quite similar for the three ions studied here: 35% for Gd, 43% for Er, and 40% for Lu. This frequency is higher than the relative frequency of BTP-like structures in all cases: 25% for Gd and Lu, and 21% for Er. Furthermore, the rapid interconversion between the two geometries is evident in the plots showing the number of equatorial oxygen atoms versus the time step of the BOMD, as depicted in Figure 7a,b. Recent classical MD simulations have reported SAP as the preferred geometry for late lanthanides.^15,32^ Our quantum-chemical-based results confirm the SAP configuration as the most likely, but with a significant probability of the BTP and a rapid transition between different structures, as shown in Table 3 and Figure 7. Moreover, the DDH-like configurations detected by applying Drew’s geometric criteria^88^ can be rotated to look very similar to either BTP or SAP, although distorted. This finding supports the conclusion that the first hydration shell does not stay at any fixed polyhedron but rapidly changes from one to another and passes through other nonregular configurations.

**Figure 5:**
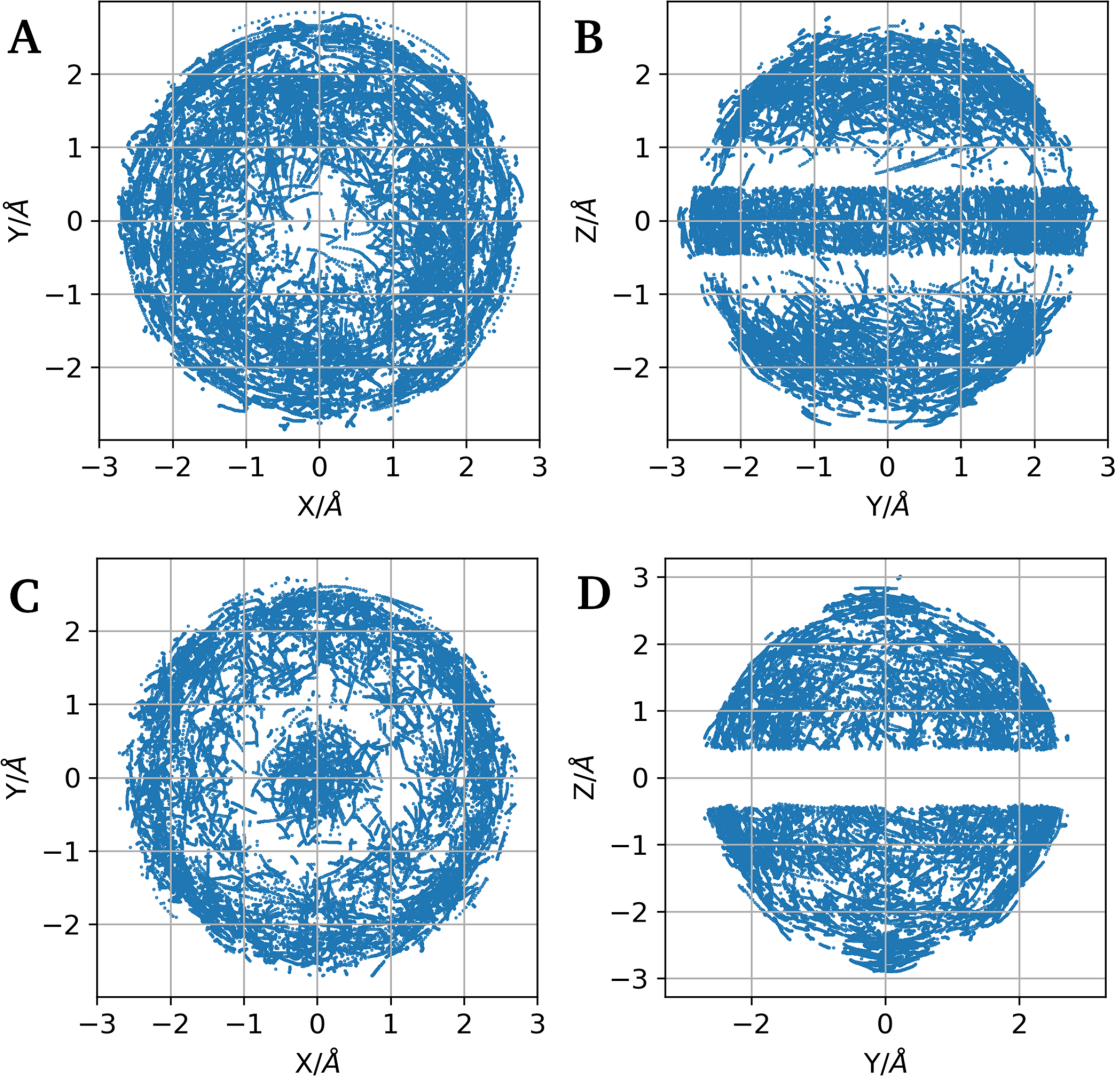
Projections of O coordinates for TTP-like (A, B) and CSAP-like (C, D) geometries of Nd^3+^ . Left panels (A, C) show projections onto the *XY* plane, and right panels (B, D) show projections onto the *YZ* plane.

**Figure 6:**
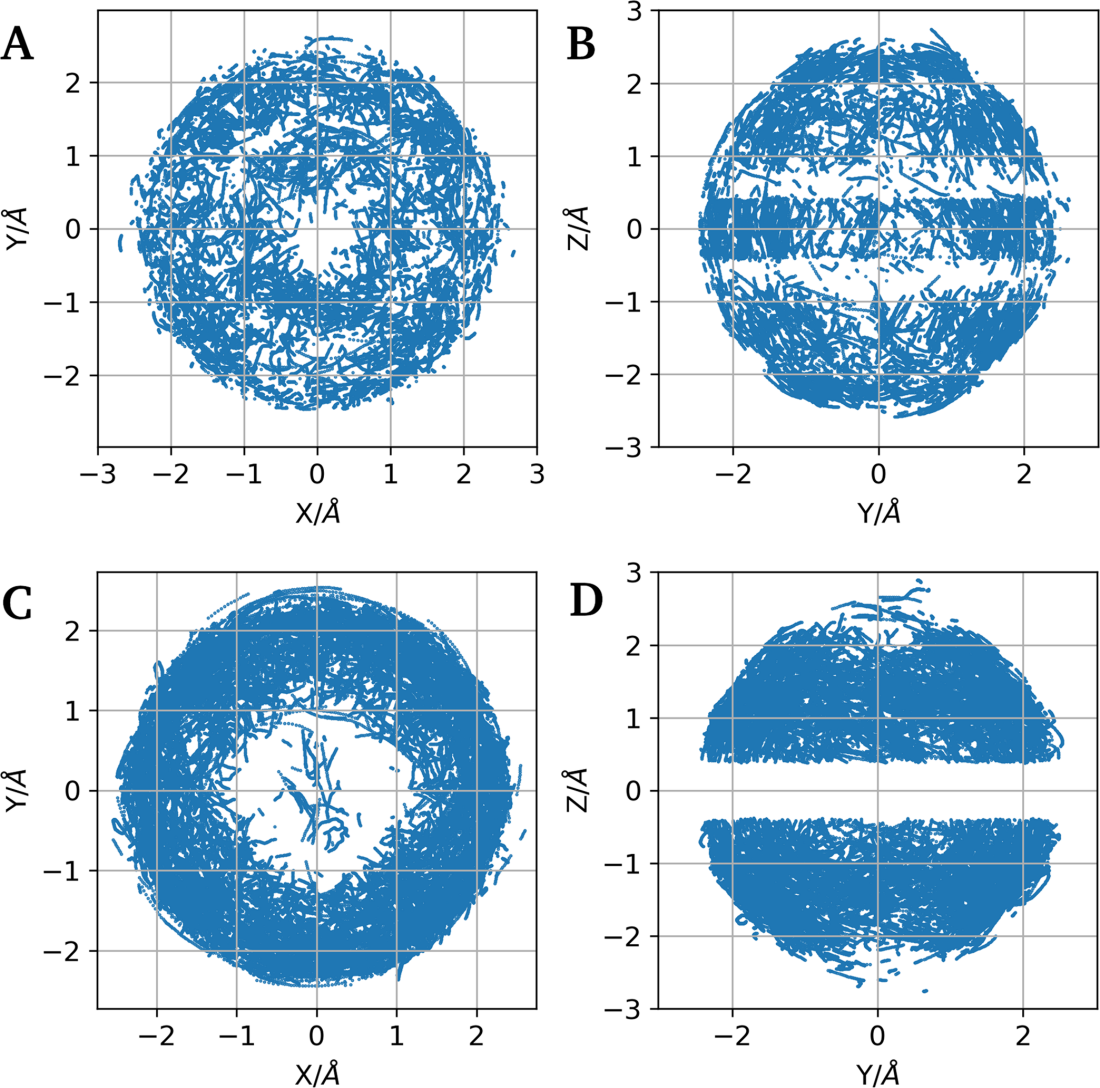
Projections of O coordinates for BTP-like (A, B) and SAP-like (C, D) geometries of Er^3+^ . Left panels (A, C) show projections onto the *XY* plane, and right panels (B, D) show projections onto the *YZ* plane.

**Figure 7:**
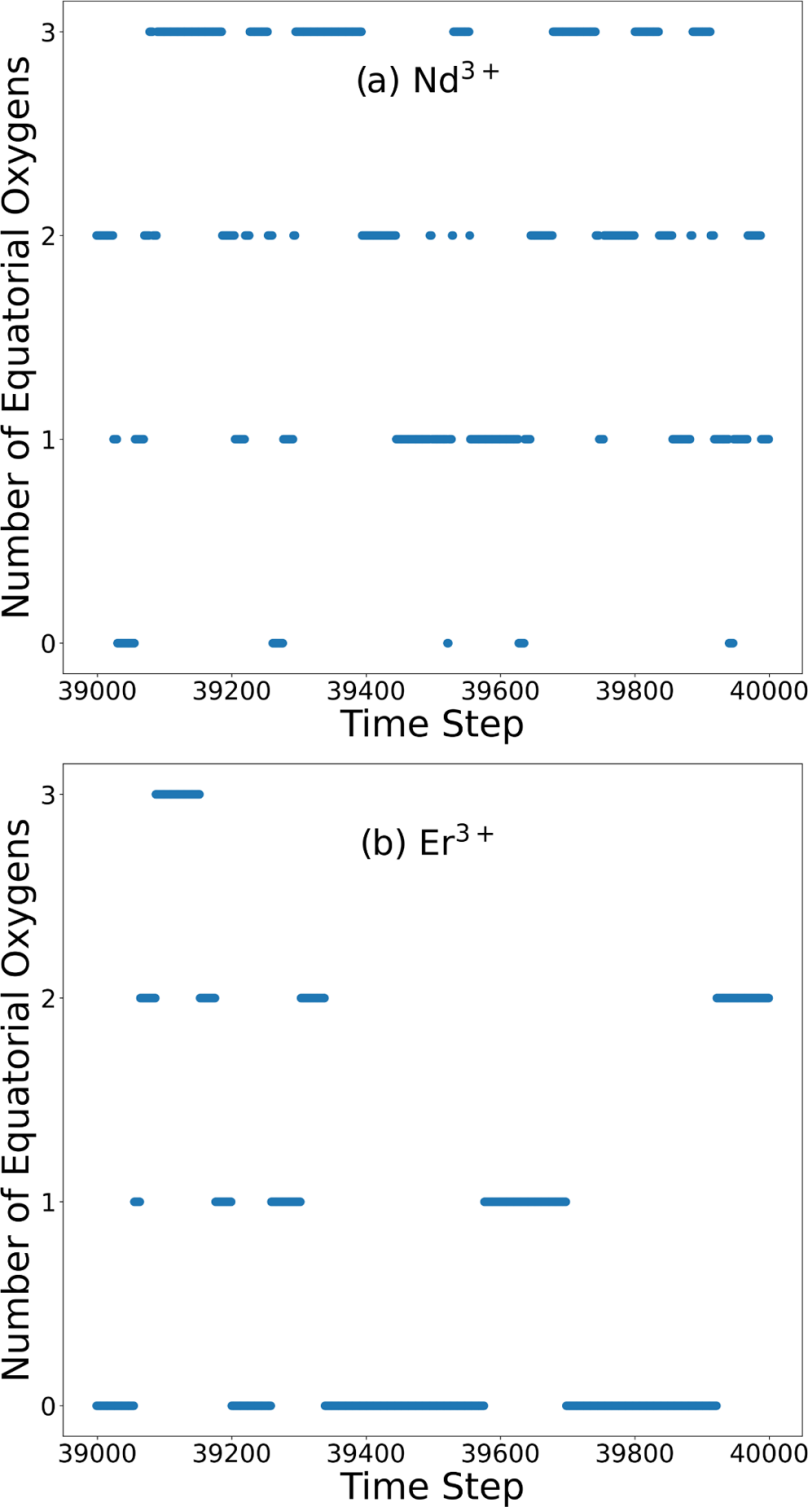
Number of equatorial oxygen atoms vs time step for the last ps of the Nd^3+^ (a) BOMD simulation and (b) for Er^3+^.

This method also enables visualization of the mobility of water molecules within the first coordination sphere, as depicted in Figures 5 and 6 for Nd^3+^ and Er^3+^, respectively. These figures show projections of coordinates from the selected geometries onto the *XY* and *YZ* planes. Figure 5A, B shows projections of coordinates from the selected geometries onto the *XY* and *YZ* planes for Nd^3+^, while Figure 6A, B presents the same projections for Er^3+^. Although the structural classification based on counting the number of equatorial oxygen atoms works effectively, the projections show significant mobility of the first hydration shell (Figure 7). There is more mobility of the nuclei than in previous studies using classical molecular dynamics by Merbach^71^ et al., from Floris and Tani,^72^ and from Villa^17^ et al. Our results align more closely with the fluxional character observed in the recent AIMD simulations by Shiery et al.^10^

To confirm the different configurations for both CNs, and as already done in similar systems,^20,68^ the first peak of each RDF was fitted with two Gaussian functions. This approach considers the likelihood of having two types of water molecules in the Ln^3+^ first hydration shell, e.g., equatorial and prismatic waters. That is different Ln^3+^-O distances are used to assess different coordination geometries, e.g., a TTP has six distances (prismatic) shorter than the remaining three (capping) thus the so-called 6 + 3 structure. The results of this fitting procedure are shown in Table 4 and Figure S15. For the early Ln^3+^ studied here (La and Nd), the CN = 9 emerges from a 5.7 + 3.3 (La) or 4.4 + 4.6 (Nd) contribution. A TTP-like configuration ideally corresponds to CNs of 6 + 3, while a CSAP configuration leads to CNs of 8 + 1. Thus, La is mostly TTP-like, while Nd represents an intermediate between TTP and CSAP. However, for the nine-membered first hydration shells, the sum of percentages of the structures resembling TTP-like and CSAP-like configurations for La^3+^ and Nd^3+^ accounts for less than 32% in both cases (Table 2). This implies that 68% of the sampled configurations do not fit the criteria for either reference polyhedron. The octa-aqua Ln^3+^ ions (Gd, Er, and Lu), associated with the reference polyhedra BTP, SAP, and the rarer DDH, exhibit CN separations of roughly 6 + 2, 8 + 0, and 4 + 4, respectively. The data in Table 4 confirm this pattern for Gd^3+^ (4 + 4) and Lu^3+^ (6 + 2). For Er^3+^, the Gaussian fit analysis indicates a CN separation of approximately 5 + 3, intermediate between the 6 + 2 and 4 + 4 separations. Again, the corresponding sums –67.5% for Gd^3+^, 73.3% for Er^3+^, and 72.9% for Lu^3+^ (Table 3)– fall short of 100%. This once again highlights that Ln^3+^ ions do not have a rigid first hydration shell.

**Table 4:** Distances of r_Ln–O_ Given in Å^a^

Ln^3+^	CN^(1,1)^	r_Ln–O_^(1,1)^	CN^(1,2)^	r_Ln–O_^(1,2)^	r_Ln–O_
La	5.65	2.58	3.35	2.70	2.62
Nd	4.42	2.49	4.58	2.59	2.52
Gd	4.06	2.39	3.94	2.47	2.42
Er	5.11	2.35	2.89	2.44	2.35
Lu	5.72	2.31	2.28	2.42	2.32

a CN^(1,*i*)^ denotes the integral of the *i*th fitting Gaussian, while r_Ln–O_^(1,*i*)^ represents its peak position.

These analyses provide valuable insights into the structural dynamics of the coordination geometries for both CN = 9 and CN = 8 ions. It suggests that these geometries are not static but exhibit a rather dynamic interconversion, more dramatically for CN = 9, challenging previous assumptions about the prevalence of certain geometries in the hydration sphere of these ions. These findings contribute to a deeper understanding of the complex behavior of lanthanide ions in solution.

### EXAFS Spectra

**Figure 8:**
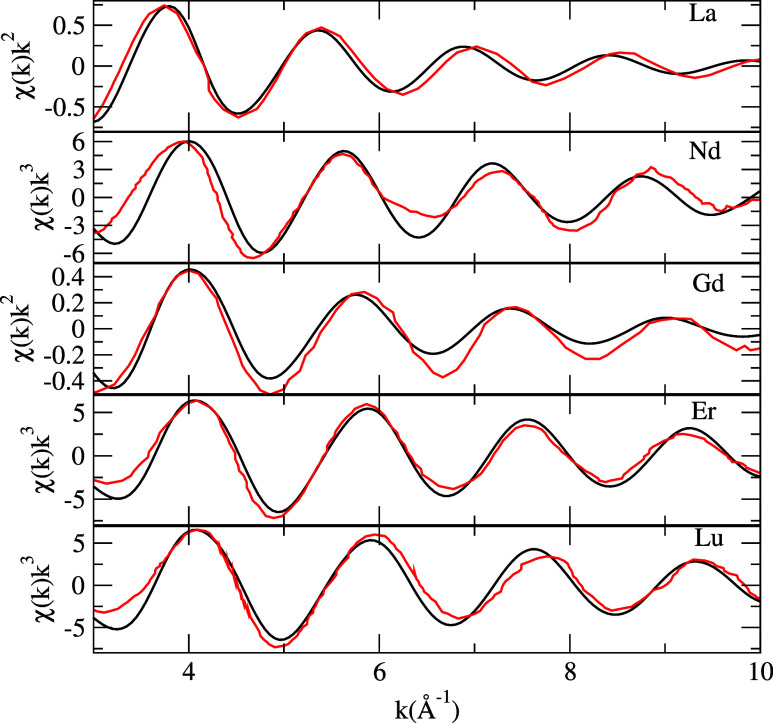
Calculated EXAFS spectra (L_1_ edge) from the BOMD simulations of hydrated La^3+^, Nd^3+^, Gd^2+^, Er^3+^, and Lu^3+^. Theoretical spectra are shown in black, and experimental spectra (red) were taken from refs (10,19,77).

We calculated the EXAFS spectra produced by our BOMD simulations as a tool to validate the structural parameters obtained (see Table 1) and to test the reliability of our simulation approach. The theoretical EXAFS spectra obtained from our BOMD simulations are shown in Figure 8 together with the corresponding experimental counterparts. A good agreement can be observed, as the theoretical spectra resemble the pattern of their experimental counterparts. The main discrepancies between theoretical and experimental spectra can be observed at around 7 Å^–1^ for Nd and Gd, and there is a slight phase shift for La and Lu, while the theoretical Er spectrum provides the best match with experimental data. Even though a perfect match was not achieved, an early attempt to calculate EXAFS spectra for Er and Nd from periodic boundary AIMD simulations has similar flaws yielding shorter than experimental peak positions and different signal phases (see Figure 3 of ref (10)). Nonetheless, in general, the position and the amplitude of the peaks in our theoretical spectra closely follow the patterns of the experimental spectra, showing that the CN and the average Ln–O distances are in good agreement with experimental measurements (Table 1). From the methodological point of view, these results show again that this kind of refined BOMD simulations with a microsolvation approach can reproduce in a precise manner the rather complex and dynamic near-solvation environment of solvent molecules around multiply charged rare-earth ions.

## Conclusions

In this work, we have performed a detailed computational study of the structural and dynamical properties of the aqueous microsolvation of Ln^3+^ ions (Ln = La, Nd, Gd, Er, and Lu) at room temperature by means of BOMD simulations. The electronic structure calculations were performed with the r^2^SCAN-3c composite method. The following conclusions arise from the results:

The composite r^2^SCAN-3c method provides overall results in agreement with experimental data concerning average Ln–O distances (MAE = 0.02 Å) of the first hydration sphere and preferred coordination numbers. The agreement of our RDFs with those of previous simulations^10,32,68^ demonstrates that our microsolvated environment accurately represents the first hydration shell structure of Ln^3+^ in the liquid state.

Zooming in on the combined distribution functions (CDFs) produced by the nonacoordinated structures, we found that at least two molecular geometries undergo noticeable and rapid rearrangements in aqueous solution, changing from one to the other. Notably, for the octacoordinated ions, our CDF findings indicate an angular distribution that while deviating from previous studies intriguingly mirrors the nonacoordinated case. This is particularly noteworthy given the conspicuous discrepancy in angular distributions between CN = 8 and CN = 9 in prior research. Our results clearly show that the octacoordinated structures swiftly change among at least three configurations (SAP, BTP, and DDH), highlighting the very dynamic nature of this octacoordinated hydration sphere in agreement with the recent proposal by Shiery et al.:^10^ there is a rather dynamical angular distribution of first-neighbor water molecules for all Ln^3+^ ions, with continuous changes in Ln–O distances and O–Ln–O angles, driven by both the inherent dynamics of the aqua ion and the thermal structural disorder in the liquid state.

On the other hand, the results of structural classification by identifying the main symmetry axis and by using geometric criteria, also reflect this fluxional characteristic:

For ions with a coordination number of CN = 9 (La^3+^ and Nd^3+^), both TTP-like and CSAP-like geometries are observed during BOMD simulations, with TTP-like geometries ocurring approximately 18% of the time and CSAP-like geometries about 13% . However, a notable 68% of the sampled configurations do not fit the criteria for either reference polyhedron. Transitioning to ions with a coordination number of CN = 8 (Gd^3+^, Er^3+^, and Lu^3+^), our analysis indicates the prevalence of both SAP-like and BTP structures. Specifically, SAP-like geometries are found with relative frequencies of 35% for Gd, 43% for Er, and 40% for Lu. Interestingly, these frequencies show only a modest increase compared to the relative frequency of BTP-like structures in all cases, which are observed at 25% for Gd and Lu and 21% for Er. Even so, the combined percentages for the latter ions fall short of 100%, again underlying the dynamic and non ideal nature of the hydration shell. These findings suggest a highly fluxional hydration environment where a substantial fraction of configurations deviate from the ideal polyhedral geometries. This further supports the notion of a dynamic angular distribution and rapid structural rearrangements within the first hydration shell. Notably, a possible pathway for a ninth water molecule to approach an octacoordinated BTP configuration with Gd^3+^, potentially transitioning to a nonacoordinated TTP will be explored in a subsequent study.

As in previous studies of other metal ions, the above results indicate that our BOMD simulations in a microsolvated environment accurately represent the near-solvation structure of ions in various environments, including aqueous solution, because the first hydration shell remains isolated from the bulk for a sufficiently long time to allow a separate study of the fast dynamics of its component molecules.

## Supporting Information Available

Conformational search details; initial seeds and xyz coordinates for La(H_2_O)_9_(H_2_O)_18_^3+^, Gd(H_2_O)_8_(H_2_O)_19_^3+^, and Lu(H_2_O)_8_(H_2_O)_16_^3+^; DFT calibration results; potential energy vs time step plots; RMSD distances; Ln–O RDFs; reference molecular geometries coordinates; CDF comparisons; Ln^3+^–O first peak RDFs with Gaussian fits; BOMD RMSD plots; and sensitivity analysis including RDFs and internal dynamics of the first hydration shell (PDF)
