# Supplementary material for: Exploring the Dynamic Coordination Sphere of Lanthanide Aqua Ions: Insights from r2SCAN-3c Composite-DFT Born–Oppenheimer Molecular Dynamics Studies
Source: ACS Omega. 2024 Dec 16;9(52):50978–91. doi: 10.1021/acsomega.4c04947 (PMC11696431; doi:10.1021/acsomega.4c04947)
Supplement: Supplementary file 1 — ao4c04947_si_001.pdf [file ao4c04947_si_001.pdf]

# Exploring the Dynamic Coordination Sphere of Lanthanide Aqua Ions: Insights from $r^2$SCAN-3c Composite-DFT Born-Oppenheimer Molecular Dynamics Studies

Emiliano Isaías Alanís-Manzano,[*,†] C. I. León-Pimentel,[‡] Laurent Maron,[¶] Alejandro Ramírez-Solís,[§] and Humberto Saint-Martin Posada[†]

†*Instituto de Ciencias Físicas, Universidad Nacional Autónoma de México, Cuernavaca Morelos 62210 México.*

‡*Departamento de Matemáticas/Fisicoquímica, Facultad de Química, Universidad Nacional Autónoma de México.*

¶*Université de Toulouse, INSA Laboratoire de Physicochimie de Nano-Objets, 135 Avenue de Rangueil, F31077 Toulouse, France*

§*Depto. de Física, Centro de Investigación en Ciencias-IICBA Universidad Autónoma del Estado de Morelos, Cuernavaca Morelos 62209 México.*

E-mail: emiliano@icf.unam.mx

# Conformational Search Details

## Initial Seeds: Optimal MP2 geometries with one hydration shell

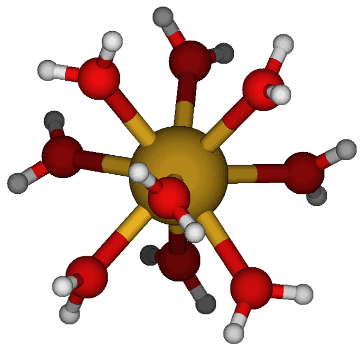

(a) Initial $La(H_2O)_9^{3+}$ seed for our conformational search.

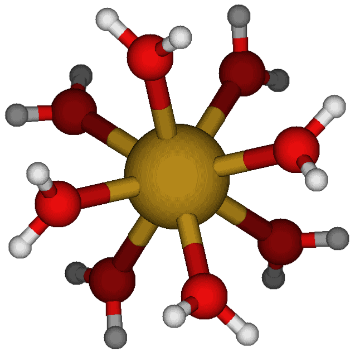

(b) Initial $Gd(H_2O)_8^{3+}$ seed for our conformational search.

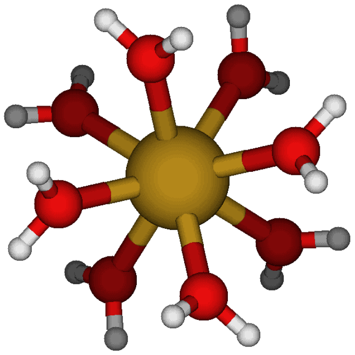

(c) Initial $Lu(H_2O)_8^{3+}$ seed for our conformational search.

Figure S1: MP2 optimal gas phase structures of Dolg *et al.* at $T = 0$ K.[1]

## $La(H_2O)_9(H_2O)_{18}^{3+}$ xyz coordinates

```
La 3.35044215515801 1.89488929017951 2.12002469291918

O 3.4068845667594 4.44824727801438 1.83752822931306

H 3.61095728989725 4.83160275115457 0.96740313976446

H 2.57309818085058 4.89677242571229 2.11218211663561

O 3.27992284437057 -0.31495778021788 0.79826283055369

H 3.59885877567559 -1.14127463303778 1.23372439219958
```

H 3.37834912182667 -0.47584645731618 -0.15120202548223

O 5.82809414517002 1.65488541387731 1.43475058600898

H 6.58648782808301 1.63916304455944 2.04750519532851

H 6.1148575461807 1.32337394910892 0.56407483803478

O 3.6408205093529 2.31339546743261 -0.41415224574736

H 4.40877604181099 1.90787366527393 -0.86235438963561

H 3.55920003744256 3.22192762559551 -0.77609481952689

O 1.02760919301923 2.49324911985278 1.15369322767517

H 0.86397765177238 3.20007229109541 0.5021166980613

H 0.30839946759713 2.58190418522146 1.80836956622996

O 1.98163929480624 2.9779248142828 4.03484922763294

H 1.00385487976341 2.93885151673884 4.00160664136837

H 2.25625465606327 3.00755989453611 4.96983734496188

O 1.52207834441373 0.29444635288042 3.15715369091581

H 1.68171463526647 0.22263189728489 4.11929134107942

H 1.46407392808807 -0.62937746525892 2.86779573910017

O 4.92837504861604 2.84466959081613 4.0366725615677

H 5.7333307490227 2.29322981394106 4.09472861288315

H 5.29380110758765 3.74431332125356 3.83384685752323

O 4.52528420282915 0.06145202252696 3.65349123710093

H 4.01495744683457 -0.11854645056778 4.47121649167991

H 4.68228237137593 -0.81117713024073 3.25057934329172

O 2.6681933612185 -0.05030704909426 5.72528601798677

H 2.44787570703057 -0.82693667019331 6.26615475002634

H 2.8330450397882 0.70525329441829 6.31595805098446

O 5.90005588025636 0.83570892407286 -1.24328751489158

H 6.55262038864268 1.05900986431942 -1.91704388829705

H 5.6044553845478 -0.07916289391866 -1.43672526220231

O 3.26913237330488 4.97938124051312 -0.98910597818528

H 2.30072493495415 4.99913381092975 -1.13922812834436

H 3.67383320630489 5.52168807161845 -1.67541679780874

O -0.71877304929127 2.91512116042022 3.38211427287544

H -1.17536744238414 3.77640473808296 3.49413151461298

H -1.34736041335447 2.23719312550315 3.65415553942467

O 3.3608629577367 2.6129990371162 6.44658707503124

H 3.46355681464999 3.12144559380769 7.25952980852192

H 4.14917790767466 2.79456326879794 5.90400704152796

O 4.20701430135203 -2.4200910721132 2.23554537266372

H 4.65518945702077 -3.11209586104127 1.72146225567104

H 3.42946676334439 -2.82094100675191 2.67184705846457

O 0.98194191007636 5.68774179886285 2.23943119938331

H 0.61396901732986 6.28933750107684 1.57364702852829

H 0.36548007543016 5.74621056698321 2.98245191982441

O 7.57533625912886 1.82621279733562 3.64089584153269

H 8.02238277640092 1.05634170617834 4.01024608922075

H 8.19342459490769 2.57426654837966 3.75603324043502

O 6.31088973623804 5.08918756168054 3.41478976204874

H 6.02189781307365 6.0056192275358 3.47415289519273

H 7.23926368428289 5.07706715342051 3.69732484462403

O 0.53079805794955 4.55492604258313 -0.90032218098467

H 0.01757771370907 5.34792356810581 -0.6376135740885

H 0.07912968580474 4.19786578832305 -1.67474580172014

O 1.7400261789286 -2.74136968652086 3.52378941347712

H 1.73496860948124 -2.84306067373493 4.49361426741222

H 1.07628834510464 -3.36540089122989 3.20592795197491

O 4.61580320818079 -1.62140265242752 -1.50282444317479

H 4.34246329872434 -1.90979573211261 -2.38195796750296

H 4.96280783983794 -2.42060748274927 -1.06476682063229

O -1.63378357857599 5.53411461217765 3.67647080615822

H -2.21785902161724 5.94592974721648 3.01392355434903

H -1.96328833118931 5.83714756553917 4.53075946682384

O 9.06518276887095 4.25585536949911 3.99841868061688

H 9.49980077152694 4.4169308120616 4.84582363840728

H 9.72121624814282 4.51261284729806 3.33773528089139

O 1.87441860213571 -2.73940840082646 6.45803795021023

H 1.05074164956115 -2.88796526196609 6.94061063326503

H 2.50379086089914 -3.34534952135625 6.87028355211719

O 5.44623166803756 -3.97001224309716 0.04409504215582

H 5.02045424454295 -4.78653435180332 -0.2486768239619

H 6.3861583949027 -4.19184651134071 0.07200900496631

O -0.62521103521255 6.91147206812838 0.10900159079162

H -1.52295680726226 6.97271010358132 0.48472239471235

H -0.49897624060832 7.72682666776426 -0.39097465903055

O -3.12245917415058 6.95291750516268 1.59300643225637

H -3.40012893394522 7.83538027997874 1.87354126378011

H -3.91818247307999 6.56402933910427 1.20650232643965

## $Gd(H_2O)_8(H_2O)_{19}^{3+}$ xyz coordinates

Gd 0.27479323631109 -0.34162668829925 -0.22335806987955

O 1.82672847442928 -1.82894256039448 -1.27645341337879

O -1.01736771869045 -1.75360839647630 -1.76461754689270

| | | | |
|---|---|---|---|
| O | -1.03887465521885 | 0.95657670769883 | -1.80779094734219 |
| O | -1.90556597229269 | -0.49370548018588 | 0.69010966588732 |
| O | -0.18215398381318 | 1.66725323214491 | 1.03406798474275 |
| O | 1.98047107671976 | -0.25367560784164 | 1.44250103398691 |
| O | 1.85892156889221 | 1.12509390055409 | -1.21149782618106 |
| O | 0.15683808654107 | -2.44229470912653 | 1.02401198671981 |
| H | 1.54068510302736 | -2.69727903702567 | -1.63244504372280 |
| H | 2.80130346785046 | -1.87665385644598 | -1.14594621058504 |
| H | -1.01685653880319 | 2.15693667115827 | 0.86794688026007 |
| H | 0.48105836612847 | 2.33815856960471 | 1.29580597508567 |
| H | 2.58097490304774 | 0.48753530522391 | 1.67857298927067 |
| H | 2.40331961120508 | -1.08305060105357 | 1.75303250223794 |
| H | -0.41624302530544 | -2.54023834960858 | 1.81515114871371 |
| H | 1.01066937356694 | -2.86002316700038 | 1.23286105602726 |
| H | -2.20820316694268 | -0.19450407619251 | 1.57822594415450 |
| H | -2.35667974331259 | -1.36211614605132 | 0.54953838778498 |
| H | -1.31496175075443 | -2.67801250597474 | -1.63459877047098 |
| H | -0.85815123113268 | -1.68031732408049 | -2.72098283314411 |
| H | 1.78638439969898 | 2.09668144464660 | -1.33271564099909 |
| H | 2.82587565142076 | 0.96653858826559 | -1.04869580881659 |
| H | -0.93699958431756 | 1.91493042577911 | -1.97942929489428 |
| H | -1.99703471004492 | 0.81508762385301 | -1.67919036890366 |
| O | 2.99564311463456 | -2.78279402825120 | 1.71353515459214 |
| H | 3.69576111782988 | -2.68587254475120 | 1.04043129685868 |
| O | -1.77506766596559 | -2.73253759748045 | 3.07970723950751 |
| H | -2.26558897075574 | -1.92264983448868 | 3.33368464628366 |
| H | 3.39241105286583 | -3.25989419222976 | 2.45218882834053 |

| | | | |
|---|---|---|---|
| H | -1.71151382672093 | -3.27519511559714 | 3.87441178889644 |
| O | 1.78123665223903 | 3.66958447978414 | 1.43433368512283 |
| H | 1.85080790626699 | 3.97180605055539 | 0.50114295597196 |
| H | 1.62689894312111 | 4.45849081503188 | 1.96833977449394 |
| O | 0.92659305306008 | -4.23966821271429 | -2.45101196044615 |
| H | 0.62891344499564 | -3.89554153679326 | -3.31354569662251 |
| O | 4.47603118311719 | -1.88957488924151 | -0.55466702910473 |
| H | 4.70251800406210 | -0.93147644574393 | -0.56649732979038 |
| H | 5.19054962092152 | -2.34082090174706 | -1.02012430020342 |
| H | 1.55654913645871 | -4.94260191332832 | -2.65384756900916 |
| O | 4.49442063181498 | 0.86079719654947 | -0.62564570726011 |
| H | 5.00121677337413 | 1.43912044200283 | -1.25177815010940 |
| O | 1.78576461352543 | 3.91898863748257 | -1.30499449385524 |
| H | 0.90685420119624 | 4.16525844897929 | -1.64742485320829 |
| O | -0.22894726046561 | -2.55835616948635 | -4.50011554647290 |
| H | 0.39791717797739 | -2.07285326796053 | -5.05134194034884 |
| O | -1.83103968188434 | -4.42208305979100 | -1.45178359137852 |
| H | -0.94508324857318 | -4.81111411926825 | -1.53205908263037 |
| O | -0.98307077245322 | 3.79624949884076 | -1.93981287657488 |
| H | -1.41054764018667 | 4.28102097155886 | -2.65694460227899 |
| O | -3.89492564165353 | 1.08942719226290 | -1.04016295032095 |
| H | -4.27850281502638 | 0.39840963620578 | -0.45935446887219 |
| H | -1.58292108594585 | 3.87001436516943 | -1.17181505009951 |
| H | -4.59937614681559 | 1.32194944861630 | -1.65753595130091 |
| H | 2.45840043545325 | 4.37561972928299 | -1.84782371279224 |
| H | 4.56147357334928 | 1.27717598886280 | 0.25230297001299 |
| H | -0.89680329080313 | -2.87316206160229 | -5.12308042661055 |

| | | | |
|---|---|---|---|
| H | -2.40147479436601 | -4.95228887292248 | -2.02199632218197 |
| O | -3.20880009417630 | -0.32247598710399 | 3.07197054457809 |
| H | -3.33647300863467 | 0.29301967812686 | 3.83117690496530 |
| O | -3.16684404744814 | -2.93839363156312 | 0.63505088363597 |
| H | -2.80996277982001 | -3.60627399821233 | 0.02468590478382 |
| H | -4.08062082587150 | -0.43206996775256 | 2.65874837163636 |
| H | -2.87563419855604 | -3.21902296246361 | 1.52184723362446 |
| O | -2.50694131068739 | 3.06044674471175 | 0.32241345878282 |
| H | -3.14582691352743 | 2.42241829968563 | -0.06996144928708 |
| H | -3.00646492432817 | 3.58753220646938 | 0.95772157068688 |
| O | -3.58911692127232 | 1.44125889414050 | 5.15623608541014 |
| H | -4.48327024135792 | 1.65642084400305 | 5.44674513659408 |
| H | -3.08143795157866 | 1.37281986119538 | 5.97298148987867 |
| O | 4.02195933477784 | 5.01177938051778 | -2.76567297646789 |
| H | 3.92428467584397 | 5.18798079346213 | -3.71094100196577 |
| H | 4.41135137360300 | 5.82282998400889 | -2.41248000467690 |
| O | 3.77508257897163 | 1.79061325371960 | 1.96863636885197 |
| H | 3.23686965342688 | 2.60679559709912 | 1.92081872639158 |
| H | 4.28488083112144 | 1.84412725796687 | 2.78608981440549 |
| O | -5.05698385603020 | -0.82704070320270 | 0.77975516233918 |
| H | -6.01995589957805 | -0.89460713984376 | 0.77832447536915 |
| H | -4.73142173560304 | -1.73087126220037 | 0.61959996410851 |
| O | 5.61505912410540 | 2.49945672766148 | -2.43649112689243 |
| H | 6.55114587167020 | 2.57008707880506 | -2.65333864377754 |
| H | 5.23935385209125 | 3.38441320980957 | -2.56387173124413 |

## $Lu(H_2O)_8(H_2O)_{16}^{3+}$ xyz coordinates

| | | | |
|---|---|---|---|
| Lu | -0.34505228574161 | -0.22142661254862 | 0.40423621232960 |
| O | 1.79062522236978 | -1.15742630633754 | 0.31768519544788 |
| O | -0.38478873901878 | -1.62805795303872 | -1.41856359804851 |
| O | -1.83105756096483 | 0.72699481691473 | -1.12934242446656 |
| O | -2.36506994393530 | -0.30191875531738 | 1.49119500682968 |
| O | -0.66847160554376 | 1.99163153257028 | 1.14255447162812 |
| O | 0.61989078313302 | 0.01975693178758 | 2.53962095124634 |
| O | 0.96100310473499 | 1.03133576886964 | -1.05054935771715 |
| O | -0.59199795869914 | -2.31420474155397 | 1.31250380117819 |
| H | 2.08511412907562 | -1.87123317529381 | -0.29110236199083 |
| H | 2.47929380458761 | -1.05900228373970 | 0.99642917239331 |
| H | -0.90716533592547 | 2.76739984695506 | 0.59453230101117 |
| H | -1.02662268329454 | 2.14964604961357 | 2.03946312166687 |
| H | 1.20799958056244 | 0.73802843642731 | 2.82975023360754 |
| H | 0.07520004462497 | -0.22379624595950 | 3.32153766974790 |
| H | -1.27128983429359 | -2.60330501594582 | 1.94892084661539 |
| H | 0.12819963602657 | -2.99805434230024 | 1.32379677949578 |
| H | -3.05183559566528 | 0.39515436195017 | 1.56915376986141 |
| H | -2.67809607287629 | -1.05499305115696 | 2.03671707195105 |
| H | -0.39409983154359 | -2.60569409966080 | -1.35449168106071 |
| H | 0.03198769495789 | -1.42749121044813 | -2.28327869483683 |
| H | 0.69983444214706 | 1.83682165762774 | -1.54076026993550 |
| H | 1.94015274243319 | 0.99799716820226 | -1.03966345349612 |
| H | -1.75356286121196 | 0.62084105955271 | -2.09706751962918 |
| H | -2.60784360825644 | 1.29953405416193 | -0.96943566206158 |
| O | -4.20833337703985 | 1.72060696194364 | 1.95356519999261 |

| | | | |
|---|---|---|---|
| H | -5.11449107493674 | 1.54148915357481 | 2.23502875216432 |
| H | -4.27488168295894 | 2.20523564266421 | 1.10423456735068 |
| O | -1.19226590082181 | -0.36421779028672 | 4.63059922375482 |
| H | -0.94991806257895 | -0.41399412172437 | 5.56411415454354 |
| H | -1.62000966999359 | 0.50662600820903 | 4.51195456115405 |
| O | 0.01354838845695 | 3.14731682269092 | -2.68596605284593 |
| H | -0.45365575946159 | 2.54622505657030 | -3.30472381531485 |
| H | 0.54622818045998 | 3.73567890907626 | -3.23568745099595 |
| O | 1.23989239419594 | -4.26680282654617 | 1.10430371515952 |
| H | 0.87367847456002 | -4.71521613901110 | 0.32286755826999 |
| H | 1.36474090878654 | -4.95104425120922 | 1.77287546498105 |
| O | 3.79529143750271 | 0.91666299578652 | -1.06525071685026 |
| H | 4.34031664068695 | 1.69934480139218 | -1.21582319960421 |
| H | 4.05942618497288 | 0.58038538059102 | -0.18227080797147 |
| O | -1.19040819856253 | 0.95089666364045 | -3.88693774331853 |
| H | -1.79732910023973 | 0.93373477561606 | -4.63771097383145 |
| H | -0.47842617890585 | 0.31334054800625 | -4.09693423887569 |
| O | 0.22691652149791 | -4.36684180704389 | -1.54304092337594 |
| H | -0.17930913682846 | -5.05689788961196 | -2.08202900311654 |
| H | 1.10926170114401 | -4.19945252084876 | -1.91512938979298 |
| O | 0.79934681595793 | -1.05295119156556 | -3.90881011204556 |
| H | 0.73122696736431 | -1.70646325179729 | -4.61660933294367 |
| H | 1.76028320474954 | -0.89795655790926 | -3.78929679142599 |
| O | -3.83685034706525 | 2.66494393591618 | -0.63097346781938 |
| H | -3.22529863221669 | 3.42725522922616 | -0.68273221711078 |
| H | -4.57865585220027 | 2.86287368026079 | -1.21684951915127 |
| O | -2.64918659668409 | -2.39487863356807 | 3.20729692650817 |

| | | | |
|---|---|---|---|
| H | -3.33764361150029 | -3.01856339579130 | 3.46807910066543 |
| H | -2.33072527447841 | -1.95280271519400 | 4.01277523889947 |
| O | -2.03787572952835 | 2.21828359367909 | 3.64982002089109 |
| H | -2.02028771847786 | 2.99055520740049 | 4.22943037130569 |
| H | -2.92907511491174 | 2.21289460747879 | 3.24580532001894 |
| O | -1.48545919850667 | 4.12640409410068 | -0.53987314571491 |
| H | -1.37197518730383 | 5.06248563704767 | -0.33265137804500 |
| H | -1.00024359346417 | 3.96995789042355 | -1.37729966220763 |
| O | 2.75679197296960 | -3.04289431392178 | -1.52008018613598 |
| H | 3.20041931227173 | -2.49761920285548 | -2.19988545233485 |
| H | 3.43462669024885 | -3.63266829936899 | -1.16611720336878 |
| O | 4.14299975975003 | -0.09351880057850 | 1.55867496003696 |
| H | 4.96026147961933 | -0.51688280525075 | 1.84999963765901 |
| H | 3.90557297191202 | 0.53559823778792 | 2.26527221079662 |
| O | 3.53741126555037 | -0.93501659421729 | -3.21447340102775 |
| H | 4.24457888593686 | -0.92706671656608 | -3.87224652389241 |
| H | 3.78267857156143 | -0.26924831729253 | -2.54086159969020 |
| O | 2.89618790090117 | 1.64895744518678 | 3.49603646701285 |
| H | 3.04976116586038 | 2.59935185690766 | 3.41558513497642 |
| H | 3.13333428406555 | 1.44727157564981 | 4.41123852089935 |

# DFT calibration: DFT-D3(BJ)/LCECP/aug-cc-pVDZ vs. r$^2$SCAN-3c (DFT = B3LYP or TPSS)

Balancing speed and precision poses a significant challenge in computational quantum chemistry. It's crucial to identify a theoretical approach that enables efficient performance of Born-Oppenheimer molecular dynamics (BOMD) simulations. Our objective is to conduct

20 ps dynamics with a time step of 0.5 fs for the integration of the nuclei's equations of motion, resulting in a total of $40{\times}10^3$ simulation steps. Since the electronic structure is computed at each time step, it's vital to keep the computation time for each step (electronic energy + gradient) within a few minutes. This is essential for conducting simulations efficiently and obtaining results within a reasonable timeframe.

Therefore, we require a theoretical framework that incorporates a suitable density functional, effective core potential (ECP), and appropriate atomic bases. Simultaneously, it's imperative that the theoretical approach is computationally efficient, allowing us to perform calculations in a reasonable time. For this endeavor we have decided that our static results of $Ln(H_2O)_9(H_2O)_{18}^{3+}$ (Ln=La, Nd and Gd) and $Ln(H_2O)_8(H_2O)_{16}^{3+}$ (Ln=Yb,Lu) cluster align with experimental data in terms of average Ln-O distances, preferred coordination numbers, and hydration free energies.

The computational approach utilized to simulate the hydration of Ln(III) ions is rooted in a thermodynamic cycle initially proposed by Goddard *et al.*[2] This model, characterized by the explicit incorporation of solvent molecules and the utilization of a polarizable continuum model, has proven effective in determining the $\Delta G_H$ values for these cations.[3,4] However, it's noteworthy that for $Ln(H_2O)_{8,9}^{3+}$ systems, the contributions from the implicit solvation model vary based on the specific type of cavity employed to encapsulate the cluster and from the solvation model itself.[3,5] In this calibration we have utilized the SMD solvation model to take into account the bulk water. The SMD model was applied to the optimized cluster structures at T = 0 K.

In Table S1, we show the typical wall times for the three methods applied to the $Gd(H2O)_8(H2O)_{19}^{3+}$ aggregate, utilizing 32 processors. Notably, the r$^2$SCAN-3c method proves to be more computationally cost-effective compared to DFT-D3(BJ)/LCECP/aug-cc-pVDZ (DFT=B3LYP or TPSS).

In Table S2, we present the performance of B3LYP, TPSS, and r$^2$SCAN3c in terms of average Ln-O distances and hydration free energy for aggregates with two explicit hydration

Table S1: Typical wall times for different methods for the $Gd(H_2O)_8(H_2O)_{19}^{3+}$ aggregate on 32 processors.

| | B3LYP-D3(BJ) | TPSS-D3(BJ) | r$^2$SCAN-3c |
|---|---|---|---|
| Energy | 6 minutes | 2 minutes | 1.5 minutes |
| Gradient | 169 s | 80 s | 30 s |

spheres. For conciseness, we introduce the notation $[Ln(H_2O)_n(H_2O)]_m^{3+}$ as Ln-n-m.

Table S2: Average Ln-O distances for the first hydration layer, along with hydration free energies, are provided for La, Gd, and Lu with two hydration spheres with the three methods.

| | | $\langle r_{\text{Ln-O}} \rangle$ | $\langle r_{\text{Ln-O}} \rangle_{exp}$ [a] | $\Delta G_H$ | $\Delta G_{exp}$ [b] | error | |
|---|---|---|---|---|---|---|---|
| | | (Å) | | (kJ/mol) | | $\langle r_{\text{Ln-O}} \rangle$ [c] (Å) | $\Delta G_H$ [d] (kJ/mol) |
| B3LYP | La-9-18 | 2.61 | 2.56 | -3266 | -3145 | 0.05 | -121 |
| | Gd-8-19 | 2.4 | 2.43 | -3463 | -3375 | -0.03 | -88 |
| | Lu-8-16 | 2.32 | 2.33 | -3638 | -3515 | -0.01 | -123 |
| | | | | | MAD[e] | 0.03 | 15 |
| | | | | | MAE[f] | 0.03 | 111 |
| TPSS | La-9-18 | 2.6 | 2.56 | -3229 | -3145 | 0.04 | -84 |
| | Gd-8-19 | 2.42 | 2.43 | -3442 | -3375 | -0.01 | -67 |
| | Lu-8-16 | 2.35 | 2.33 | -3626 | -3515 | 0.02 | -111 |
| | | | | | MAD | 0.02 | 16 |
| | | | | | MAE | 0.03 | 87 |
| r$^2$SCAN-3c | La-9-18 | 2.61 | 2.56 | -3264 | -3145 | 0.05 | -119 |
| | Gd-8-19 | 2.41 | 2.43 | -3592 | -3375 | -0.02 | -217 |
| | Lu-8-16 | 2.32 | 2.33 | -3758 | -3515 | -0.01 | -243 |
| | | | | | MAD | 0.03 | 49 |
| | | | | | MAE | 0.03 | 193 |

[a] Experimental Ln-O distances are obtained by averaging all the experimental figures from the references[6–12]

[b] Obtained from Marcus[13]

[c] Evaluated as $\langle r_{\text{Ln-O}} \rangle$ - $\langle r_{\text{Ln-O}} \rangle_{exp}$

[d] Evaluated as $\Delta G_H$ - $\Delta G_{exp}$

[e] MAD stands for Mean Absolute Deviation

[f] MAE stands for Mean Absolute Error

The average Ln-O distances to the first hydration sphere obtained using the three methods are in agreement with experimental values, with a maximum mean absolute error (MAE) of 0.03. As for the $\Delta G_H$ values, the largest MAE is obtained with the composite method. However, as mentioned earlier, the error of 243 kJ/mol for Lu-8-16 represents approxi-

mately 6% of the total hydration free energy. For B3LYP and TPSS, the error is 3% of this value. It is evident that the $r^2$SCAN-3c method performs well in terms of both the average Ln-O distances and the hydration free energy ($\Delta G_H$), and it is computationally more cost-effective than the DFT-D3(BJ)/LCECP/aug-cc-pVDZ approach (where DFT can be B3LYP or TPSS).

Table S3: Free energies of reaction (1) (kcal/mol) for La, Gd, and Lu in aqueous solution using the SMD model, including all its components, for the three different DFT-D3(BJ)/LCECP/aug-cc-pVDZ methods (DFT=B3LYP or TPSS) and $r^2$SCAN-3c.

| Ln | $\Delta G$ | $\Delta\Delta G_{ENP}$ | $\Delta\Delta G_{CDS}$ | $\Delta\Delta G_{SMD}$ | $\Delta G_{rxn}$ |
|---|---|---|---|---|---|
| | | | B3LYP | | |
| La | -7.51 | 9.11 | -0.04 | 9.07 | 1.56 |
| Gd | -1.14 | 0.69 | 0.04 | 0.73 | -0.42 |
| Lu | 6.14 | 1.01 | 1.10 | 2.11 | 8.25 |
| | | | TPSS | | |
| La | -7.54 | 9.84 | -0.1 | 9.74 | 2.21 |
| Gd | 2.94 | -1.29 | -0.04 | -1.33 | 1.61 |
| Lu | 11.65 | -1.75 | 0.71 | -1.04 | 10.6 |
| | | | $r^2$SCAN-3c | | |
| La | -8.23 | 3.81 | 0.6 | 4.41 | -3.82 |
| Gd | 0.43 | 1.64 | 0.72 | 2.36 | 2.79 |
| Lu | 3.61 | 3.61 | 0.77 | 4.38 | 7.98 |

Regarding the spontaneity of the water molecule transfer reaction, Table S3 displays the $\Delta G_{rxn}$ along with its components for the different methods. To achieve proper alignment with the preferred coordination numbers (NC) of $Ln^{3+}$ ions, we expect the free energy of reaction ($\Delta G_{rxn}$) to be highly negative and significant for La, small in absolute value for Gd, and positive and substantial for Lu. Upon analyzing the results, it is evident that we obtain the expected outcomes except for La, regardless of the method used. For La, we observe a considerably large contribution, $\Delta\Delta G_{CDS}$, compared to the expected value. This could be attributed to the type of cavity used or the possibility that we are not considering the true global minimum. If we momentarily assume, based on findings for Ce(III) by Dinescu and Clark, that two explicit hydration spheres suffice to account for solvent effects in the water transfer reaction (i.e., $\Delta G \sim \Delta G_{rxn}$), we obtain the preferred NC regardless of the method

used. For Gd and Lu, we find that $\Delta G = \Delta G_{rxn}$ within a maximum deviation of 2 kcal/mol.

$$[\mathrm{Ln(H_2O)_{n-1}(H_2O)_m}]^{3+}(\mathrm{aq}) \longrightarrow [\mathrm{Ln(H_2O)_n(H_2O)_{m-1}}]^{3+}(\mathrm{aq}) \quad (1)$$

Despite the challenges in calculating thermodynamic properties, the $r^2$SCAN-3c method demonstrates good performance in reproducing experimental Ln-O distances. Furthermore, this method is comparable to the DFT-D3(BJ)/LCECP/aug-cc-pVDZ approach (DFT=B3LYP or TPSS) in terms of accuracy but offers computational efficiency, allowing us to conduct 20 ps dynamics. It's important to note that the majority of the error in thermodynamic properties arises from the use of the SMD model rather than electronic structure description.

Table S4: Average Ln-O distances for the first hydration layer, along with the hydration free energies for Ln (Ln=La-Nd, Eu-Tb, Yb, and Lu) using the $r^2$SCAN-3c method.

| | $\langle r_{\text{Ln-O}} \rangle$ | $\langle r_{\text{Ln-O}} \rangle_{exp}$ [a] | $\Delta G_H$ | $\Delta G_{exp}$ [b] | error | |
|---|---|---|---|---|---|---|
| | (Å) | | (kJ/mol) | | $\langle r_{\text{Ln-O}} \rangle$ [c] (Å) | $\Delta G_H$ [d] (kJ/mol) |
| | $\langle r_{\text{Ln-O}} \rangle$ | $\langle r_{\text{Ln-O}} \rangle_{exp}$ | $\Delta G_H$ | $\Delta G_{exp}$ | $\langle r_{\text{Ln-O}} \rangle$ | $\Delta G_H$ |
| La-9-18 | 2.61 | 2.56 | -3264 | -3145 | 0.05 | -119 |
| Ce-9-18 | 2.57 | 2.54 | -3370 | -3200 | 0.03 | -170 |
| Pr-9-18 | 2.55 | 2.53 | -3409 | -3245 | 0.02 | -164 |
| Nd-9-18 | 2.54 | 2.51 | -3487 | -3280 | 0.03 | -207 |
| Sm-9-18 | 2.50 | 2.47 | -3527 | -3325 | 0.03 | -202 |
| Eu-8-19 | 2.43 | 2.44 | -3534 | -3360 | 0.04 | -174 |
| Gd-8-19 | 2.41 | 2.43 | -3592 | -3375 | -0.02 | -219 |
| Tb-8-19 | 2.41 | 2.41 | -3578 | -3400 | 0 | -178 |
| Yb-8-16 | 2.33 | 2.34 | -3686 | -3570 | -0.01 | -116 |
| Lu-8-16 | 2.32 | 2.33 | -3758 | -3515 | -0.01 | -243 |
| | | | | MAD[e] | 0.02 | 31 |
| | | | | MAE[f] | 0.02 | 179 |

[a] Experimental Ln-O distances are obtained by averaging all the experimental figures from the references [6–12]

[b] Obtained from Marcus [13]

[c] Evaluated as $\langle r_{\text{Ln-O}} \rangle$ - $\langle r_{\text{Ln-O}} \rangle_{exp}$

[d] Evaluated as $\Delta G_H$ - $\Delta G_{exp}$

[e] MAD stands for Mean Absolute Deviation

[f] MAE stands for Mean Absolute Error

It is worthwhile to investigate the performance of $r^2$SCAN-3c for a broader range of lanthanides. Table S4 displays the average Ln-O distances for the first hydration layer and

the associated hydration free energies for 10 out of the 15 lanthanides (Ln=La-Nd, Sm-Tb, Yb, and Lu) using the r$^2$SCAN-3c method. The average Ln-O distances for the first hydration sphere are reproduced with a precision of up to 0.05 Å compared to experimental values, resulting in a mean absolute error (MAE) of 0.02 Å for the set of 10 hydrated lanthanides. Regarding hydration free energies ($\Delta G_H$), a maximum error of -121 kJ/mol is observed for Lu, with an overall MAE of 179 kJ/mol for the entire set under study.

Table S5: Free energies of reaction (1) (kcal/mol) at the r$^2$SCAN-3c level for the set of 10 Ln in aqueous solution using the SMD model, including all its components.

| Ln | $\Delta G$ | $\Delta\Delta G_{ENP}$ | $\Delta\Delta G_{CDS}$ | $\Delta\Delta G_{tot}$ | $\Delta G_{rxn}$ |
|---|---|---|---|---|---|
| | | r$^2$SCAN-3c | | | |
| La | -8.23 | 3.81 | 0.6 | 4.41 | -3.82 |
| Ce | -2.26 | 2.71 | 0.39 | 3.1 | 0.83 |
| Pr | -15.11 | 2.92 | 0.37 | 3.29 | -11.83 |
| Nd | -1.5 | 3.3 | 0.39 | 3.69 | 2.19 |
| Sm | -1.6 | 1.83 | 0.7 | 2.53 | 0.93 |
| Eu | 3.59 | -3.48 | 0.78 | -2.7 | 0.89 |
| Gd | 0.43 | 1.64 | 0.72 | 2.36 | 2.79 |
| Tb | 0.45 | 1.68 | 0.7 | 2.38 | 2.82 |
| Yb | 2.87 | 3.45 | 0.86 | 4.31 | 7.18 |
| Lu | 3.61 | 3.61 | 0.77 | 4.38 | 7.98 |

The results of $\Delta G_{rxn}$ for reaction (1) for the set of 10 Ln are presented in Table S5. A common trend is observed across all lanthanides: an approximate $\Delta\Delta G_{CDS}$ contribution of 1 kcal/mol. While this contribution might be insignificant for reactions with large $\Delta G$, it can hold significance in reactions with small $\Delta G$, as seen in the cases of Nd, Sm, Gd, and Yb. Moreover, the $\Delta\Delta G_{tot}$ contribution doesn't nullify and indeed contributes to the free energy of the reaction.

One advantage of Born-Oppenheimer molecular dynamics (BOMD) is that starting from a representative structure of the system with a couple of tens of water molecules, a valid sampling of configurations consistent with the desired temperature is obtained.

After conducting a thorough comparison of the results obtained from DFT-D3(BJ)/aug-cc-pVDZ/LCECP (DFT=B3LYP and TPSS) and r$^2$SCAN-3c, we have concluded that the

composite method, $r^2$SCAN-3c, strikes an excellent balance between precision and speed, particularly concerning average Ln-O distances (MAE=0.02 Å), hydration free energies (MAE=179 kJ/mol), and preferred coordination numbers.

## Potential Energy vs. time step plots of the BOMD simulations

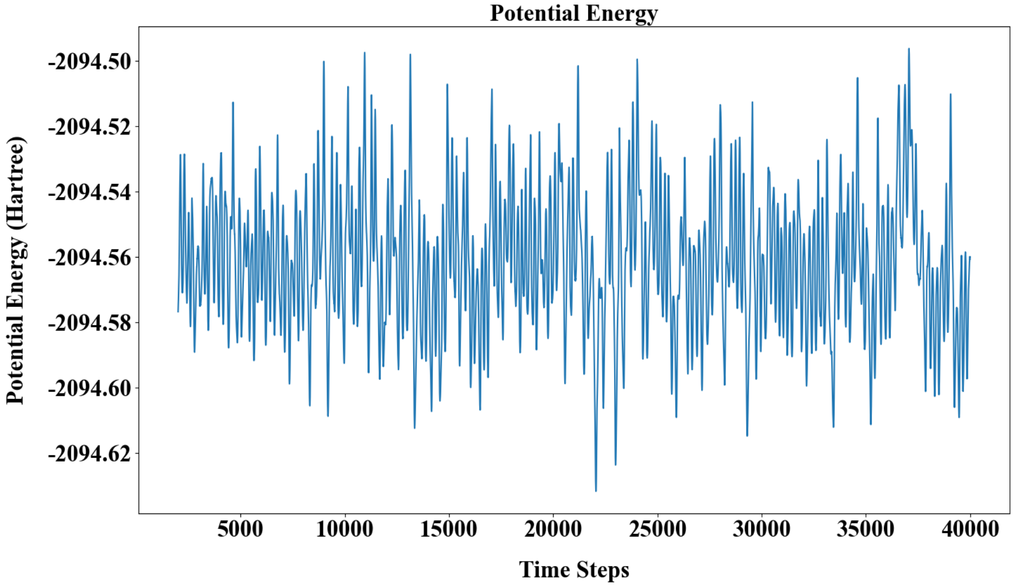


Figure S2: Potential energy of the $La^{3+}$ BOMD simulation along the equilibrated frames.

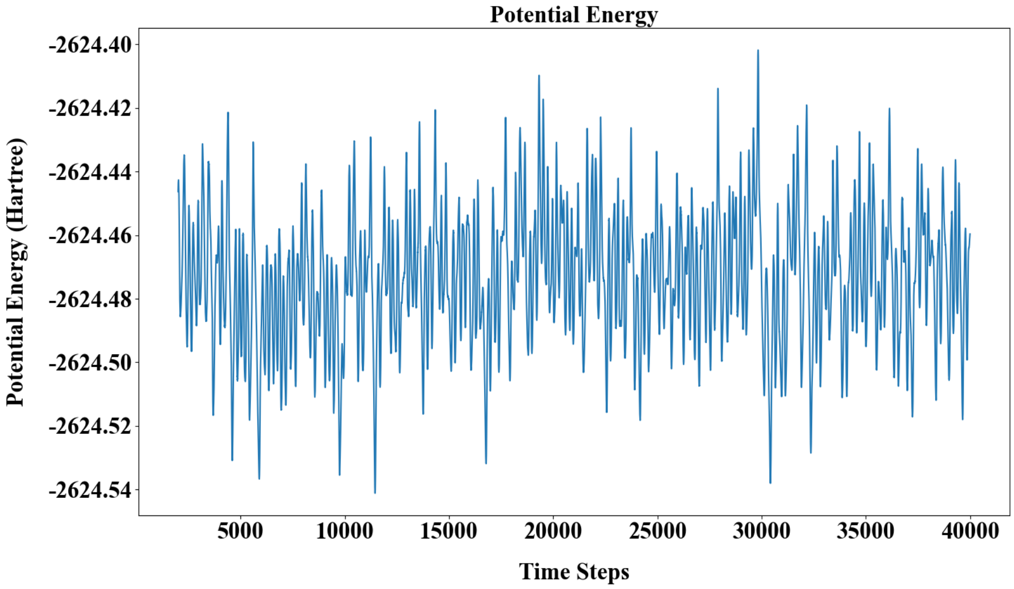


Figure S3: Potential energy of the $Nd^{3+}$ BOMD simulation along the equilibrated frames.

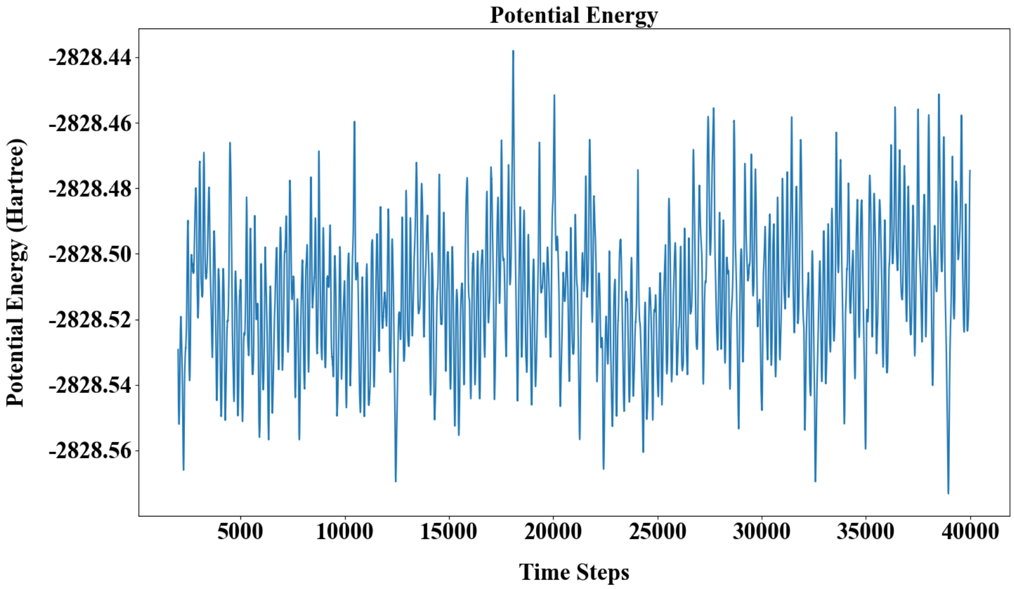


Figure S4: Potential energy of the $Gd^{3+}$ BOMD simulation along the equilibrated frames.

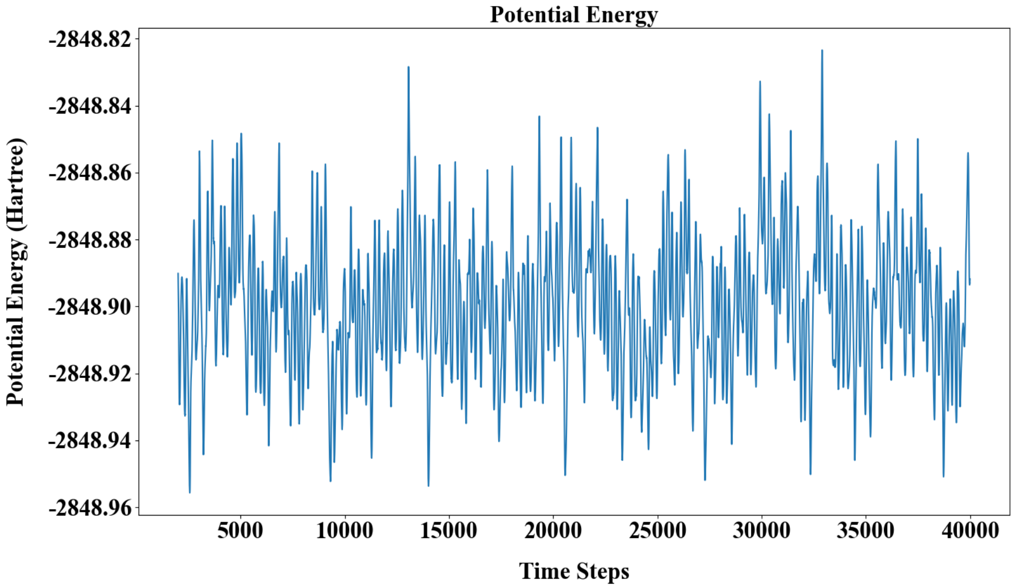


Figure S5: Potential energy of the $Er^{3+}$ BOMD simulation along the equilibrated frames.

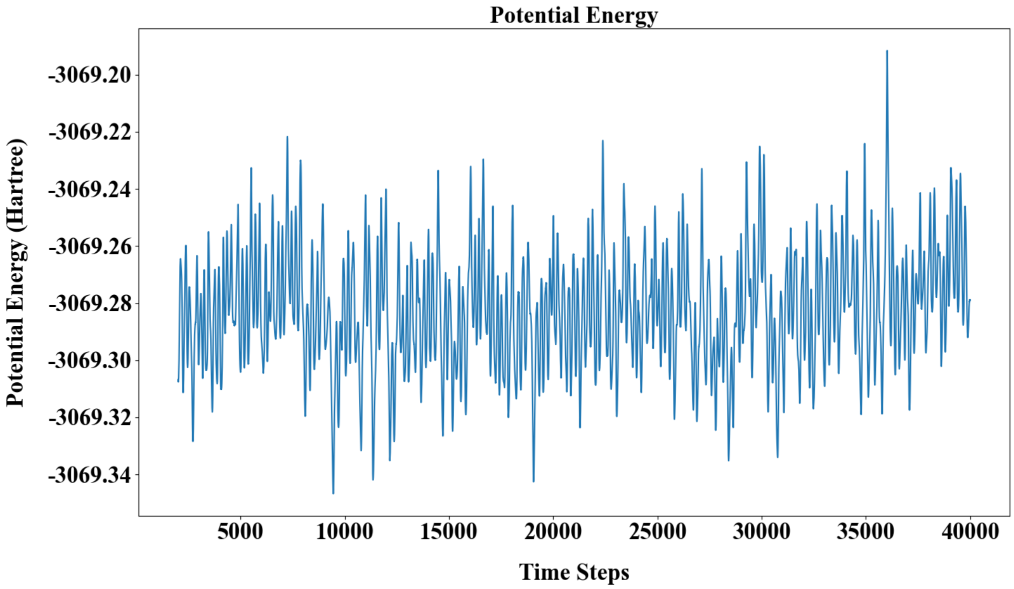


Figure S6: Potential energy of the $Lu^{3+}$ BOMD simulation along the equilibrated frames.

# Root Mean Square Deviation (RMSD) distance between the first coordination shell of each BOMD frame and the reference molecular geometries.

Consistent with the approach outlined by Shiery *et al.*,[14] we computed the root mean square deviation (RMSD) distance between the first coordination shell of each BOMD frame and the reference molecular geometries. This allowed us to assess how closely each lanthanide ion resembles a particular reference molecular geometry. The frame-averaged RMSD values with respect to the molecular reference geometries for the five $Ln^{3+}$ studied here are presented in table S6. Our results for La and Nd show RMSD values for the CSQU geometry ($\sim$ 0.80), which are significantly higher than those for CSAP and TTP ($\sim$ 0.40). This is expected, as visual analysis of some frames indicates that the first coordination sphere does not closely resemble the CSQU geometry. To the best of our knowledge, previous simulations had not reported a CSQU geometry. Initially, Shiery *et al.* reported RMSD values for CSQU comparable (within the standard deviation) to those of CSAP and TTP for CN=9. However, a recent correction to their work now reports a higher RMSD for CSQU, aligning with our findings. Our RMSD analysis for the nona-coordinated lanthanide ions supports the conclusion that the CSQU geometry should be discarded, with TTP and CSAP being the

Table S6: Average RMSD between the eight- and nine-coordinated $Ln^{3+}$ ions and the reference molecular geometries.

| | Average RMSD ± standard deviation (Å) | | |
|---|---|---|---|
| | CSAP | CSQU | TTP |
| La | 0.36 ± 0.07 | 0.88 ± 0.08 | 0.35 ± 0.08 |
| Nd | 0.33 ± 0.06 | 0.85 ± 0.08 | 0.34 ± 0.09 |
| | DDH | SAP | BTP |
| Gd | 0.31 ± 0.07 | 0.36 ± 0.09 | 0.32 ± 0.05 |
| Er | 0.29 ± 0.07 | 0.32 ± 0.09 | 0.32 ± 0.05 |
| Lu | 0.30 ± 0.06 | 0.30 ± 0.08 | 0.30 ± 0.05 |

predominant configurations

The octa-coordinated lanthanide ions exhibit RMSD values that are noticeably different from zero, indicating a lack of close fit with any of the reference molecular geometries. The highest RMSD value is observed for the SQU geometry (approximately 0.80) among the three 8-coordinated ions studied here. This value is considerably higher than the one initially reported by Shiery *et al.* (approximately 0.50), which has since been corrected to approximately 0.80. Moreover, previous simulations demonstrated that a rearrangement from SAP to this energetically unfavorable SQU geometry does not occur. The AIMD simulations conducted by Shiery *et al.* initially yielded SQU RMSD values comparable to those of the other reference molecular geometries (within the standard deviation). However, with their recent correction, their SQU RMSD values now align with ours. Consequently, we have decided not to present the RMSD values for the SQU geometries, as they remain consistently twice as large as those for the other molecular geometries (DDH, SAP, and BTP). Thus, the SQU polyhedron will not be further considered. For the DDH, SAP, and BTP molecular geometries, the RMSD values are quite similar, around 0.30, for the three octa-aqua $Ln^{3+}$ ions studied here. This suggests a relatively disordered first coordination sphere, precluding the clear identification of a single molecular geometry

## Ln-O RDFs from BOMD simulations

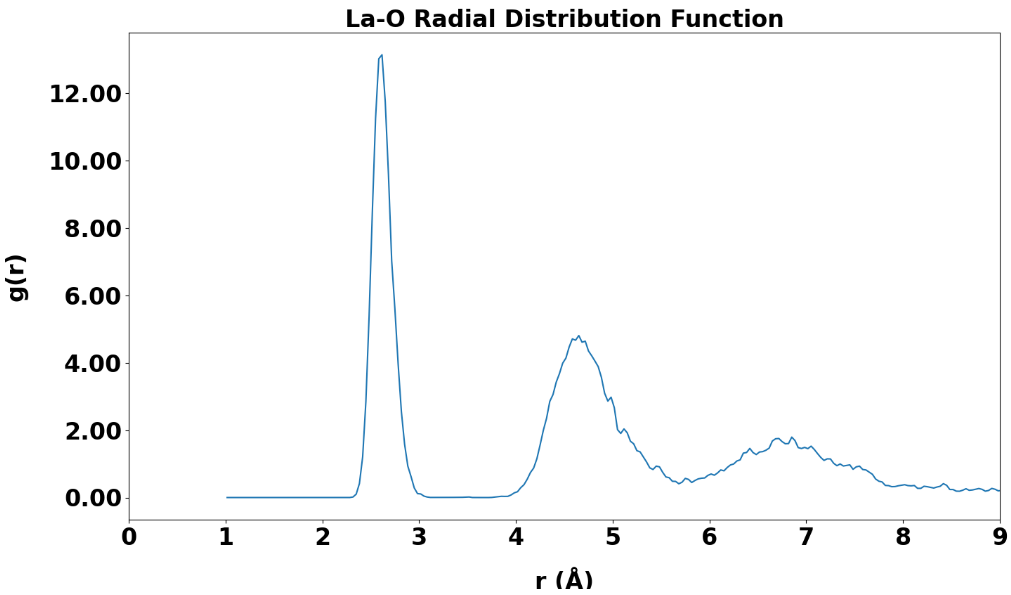


Figure S7: La-O radial distribution function from our BOMD simulation.

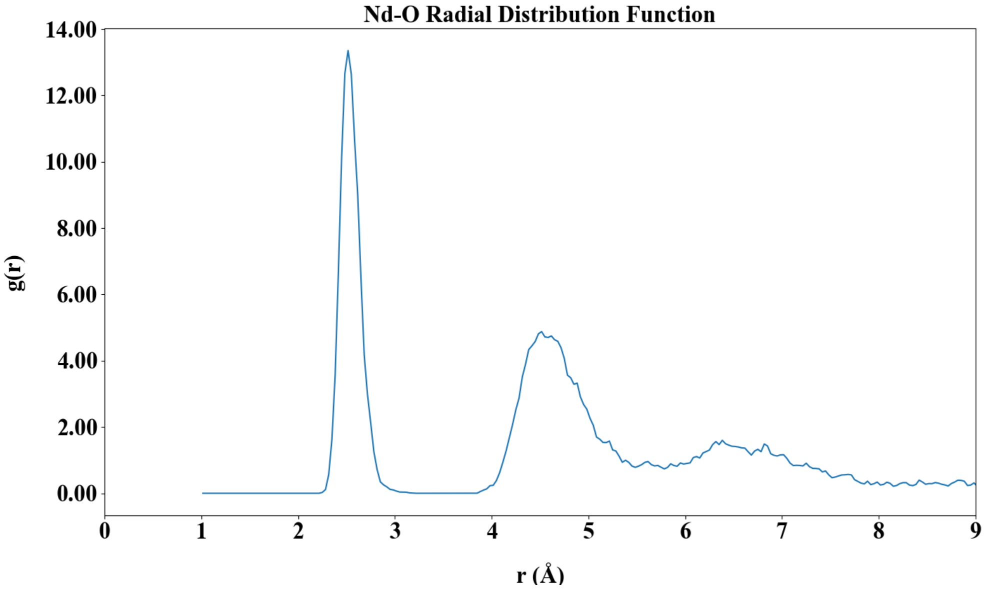


Figure S8: Nd-O radial distribution function from our BOMD simulation.

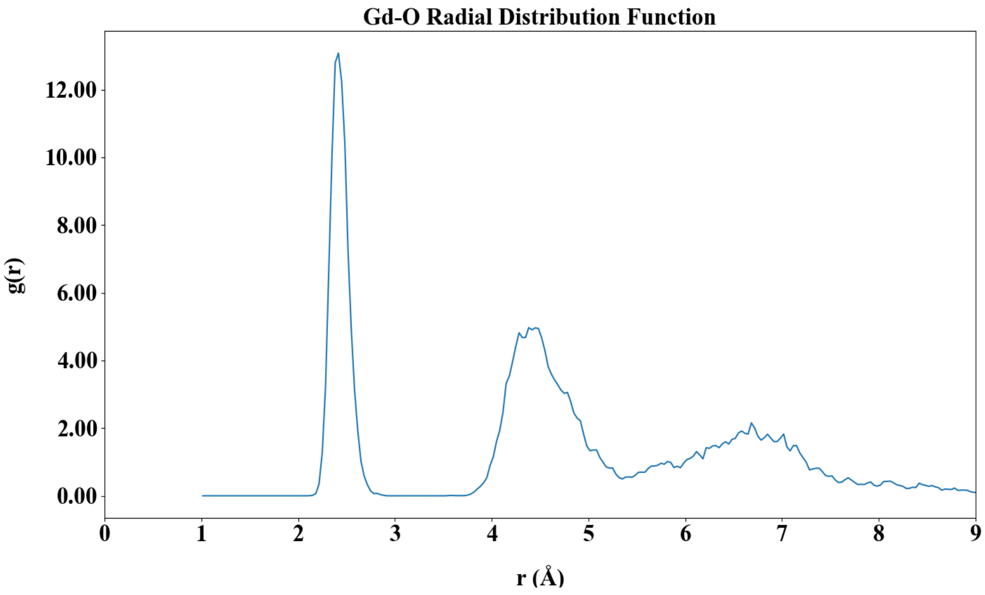


Figure S9: Gd-O radial distribution function from our BOMD simulation.

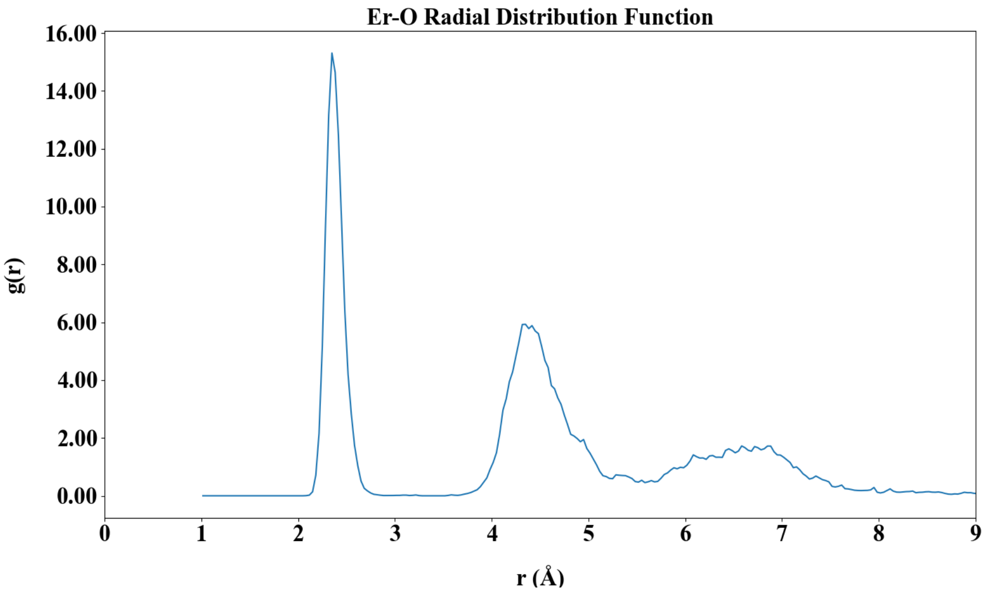


Figure S10: Er-O radial distribution function from our BOMD simulation.

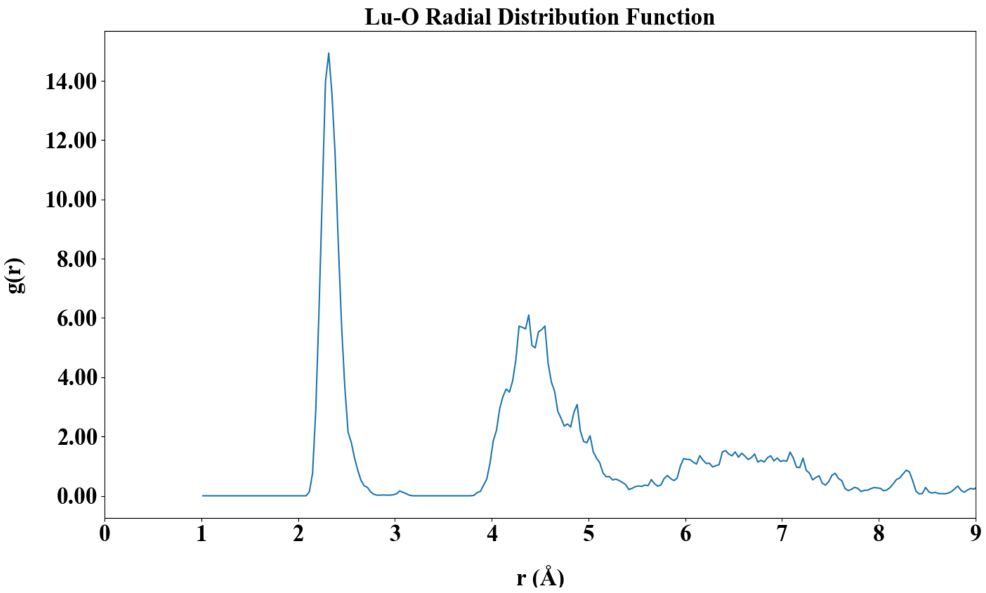


Figure S11: Lu-O radial distribution function from our BOMD simulation.

# Coordinates of the reference molecular geometries

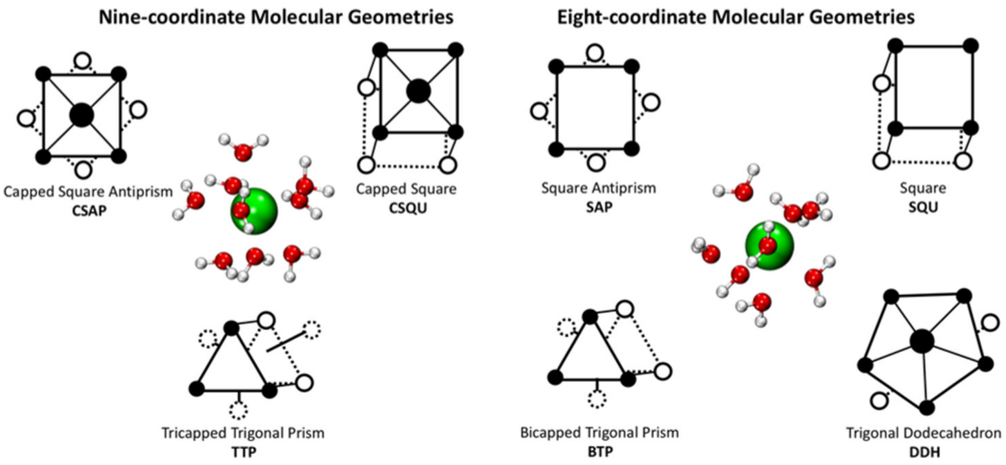


Figure S12: The image depicts the molecular geometries for nona- and octa-coordinated $Ln^{3+}$ ions. $Ln^{3+}$ ions (green); oxygen, and hydrogen are colored red and white, respectively. Black and white figures represent reference molecular geometries, where the circles show the oxygen atom positions. Reprinted with permission from *Inorg. Chem.* 2021, 60, 5, 3117-3130. Copyright © 2021 American Chemical Society

**Model tricapped-trigonal-prismatic atomic Coordinates of the $Nd(H_2O)_9^{3+}$ complex.**

| Atom | x (Å) | y (Å) | z (Å) |
|---|---|---|---|
| Nd | 0.000000 | 0.000000 | 0.000000 |
| O | 0.000000 | 1.879876 | 1.628020 |
| O | 0.000000 | 1.879876 | -1.628020 |
| O | -1.628020 | -0.939938 | 1.628020 |
| O | -1.628020 | -0.939938 | -1.628020 |
| O | 1.628020 | -0.939938 | 1.628020 |
| O | 1.628020 | -0.939938 | -1.628020 |
| O | 2.239815 | 1.293158 | 0.000000 |
| O | -2.239815 | 1.293158 | 0.000000 |
| O | 0.000000 | -2.586316 | 0.000000 |

**Model capped-square-antiprismatic atomic Coordinates of the $Nd(H_2O)_9^{3+}$ complex.**

| Atom | x (Å) | y (Å) | z (Å) |
|---|---|---|---|
| Nd | 0.000000 | 0.000000 | 0.000000 |
| O | 2.156438 | 1.282226 | 0.000000 |
| O | -2.156438 | 1.282226 | 0.000000 |
| O | 0.000000 | 1.282226 | 2.156438 |
| O | 0.000000 | 1.282226 | -2.156438 |
| O | 1.524832 | -1.282226 | 1.524832 |
| O | -1.524832 | -1.282226 | 1.524832 |
| O | 1.524832 | -1.282226 | -1.524832 |
| O | -1.524832 | -1.282226 | -1.524832 |
| O | 0.000000 | 2.609204 | 0.000000 |

### Model bicapped-trigonal-prismatic atomic Coordinates of the $Er(H_2O)_8^{3+}$ complex.

| Atom | $x$(Å) | $y$(Å) | $z$(Å) |
|---|---|---|---|
| Nd | 0.000000 | 0.000000 | 0.000000 |
| O | 0.000000 | 1.758845 | 1.523204 |
| O | 0.000000 | 1.758845 | -1.523204 |
| O | -1.523204 | -0.879422 | 1.523204 |
| O | -1.523204 | -0.879422 | -1.523204 |
| O | 1.523204 | -0.879422 | 1.523204 |
| O | 1.523204 | -0.879422 | -1.523204 |
| O | 2.095610 | 1.209901 | 0.000000 |
| O | -2.095610 | 1.209901 | 0.000000 |

## CDF

### Comparison of Cantu's reference molecular geometries

In the case of TTP, CSAP, and BTP polyhedra, two characteristic $Ln^{3+}$-O distances are observed. As initially proposed by Cantu *et al.*,[15] the optimal reference geometries for these polyhedra are designed to align the average of all ion-vertex distances with the peak value of the first $g(r)$ peak in the corresponding RDF resulting from the simulation. Diverging from Cantu's approach, we adapted their code[15] to generate our reference TTP, CSAP, and BTP polyhedra. This adaptation ensured a specific ratio $\frac{r_{\text{Ln-O(capping)}}}{r_{\text{Ln-O(prismatic)}}}$ of 1.04, consistent with both experimental neutron diffraction results[16] and prior simulations.[17]

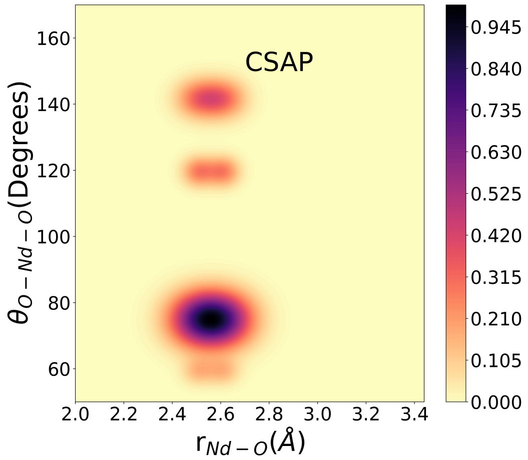


(a) Theoretical CDF evaluated for a CSAP model of the $Nd(H_2O)_9^{3+}$ complex.

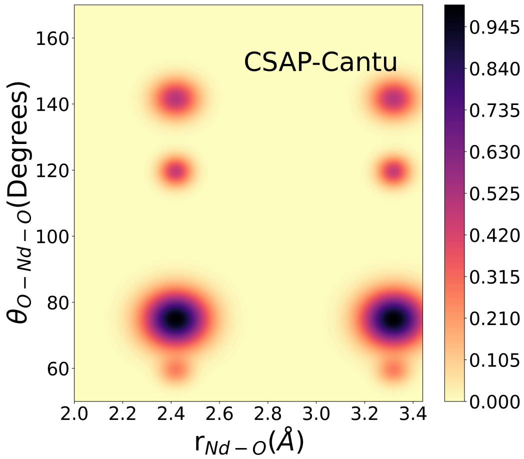


(b) Theoretical CDF evaluated for the Cantu's CSAP model of the $Nd(H_2O)_9^{3+}$ complex

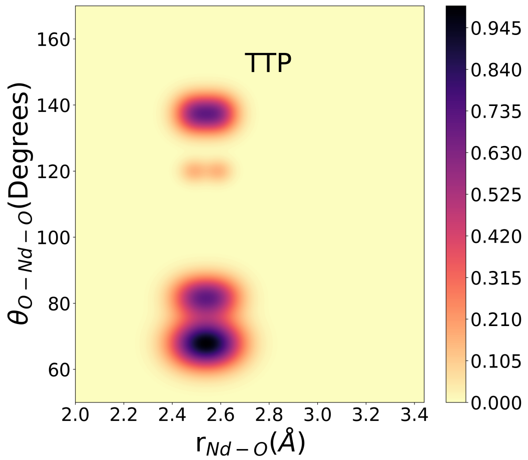


(c) Theoretical CDF evaluated for a TTP model of the $Nd(H_2O)_9^{3+}$ complex.

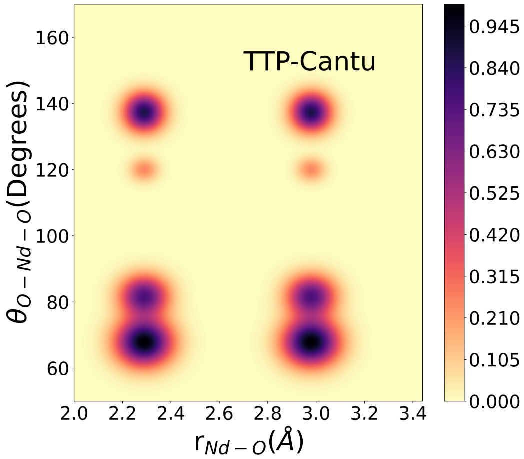


(d) Theoretical CDF evaluated for the Cantu's TTP model of the $Nd(H_2O)_9^{3+}$ complex.

Figure S13: Comparison of the theoretical CDF's of the different models of 9-coordinate reference polyhedrons.

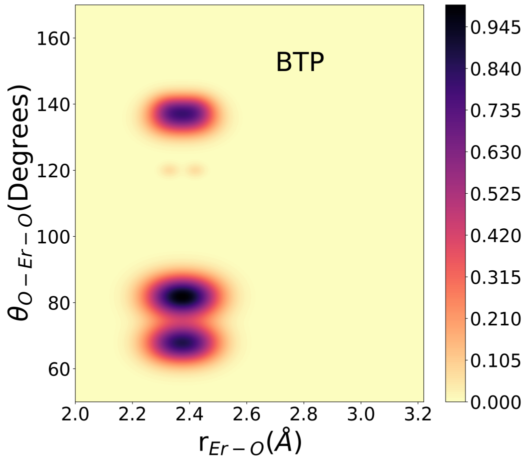


(a) Theoretical CDF evaluated for our BTP model of the $Er(H_2O)_8^{3+}$ complex.

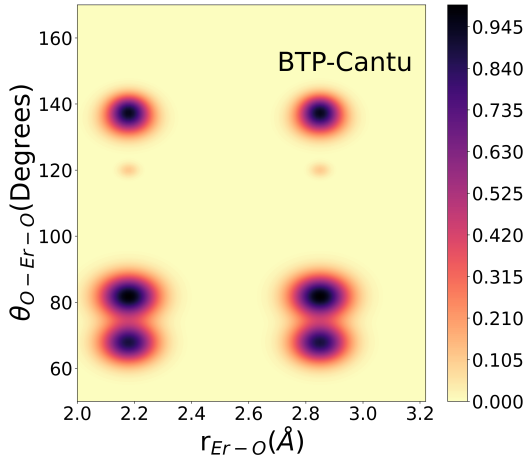


(b) Theoretical CDF evaluated for the Cantu's TTP model of the $Er(H_2O)_8^{3+}$ complex.

Figure S14: Comparison of the theoretical CDF's of the two BTP models of the 8-coordinate reference polyhedrons.

# $Ln^{3+}$-O first peak RDFs with the fitting Gaussian curves

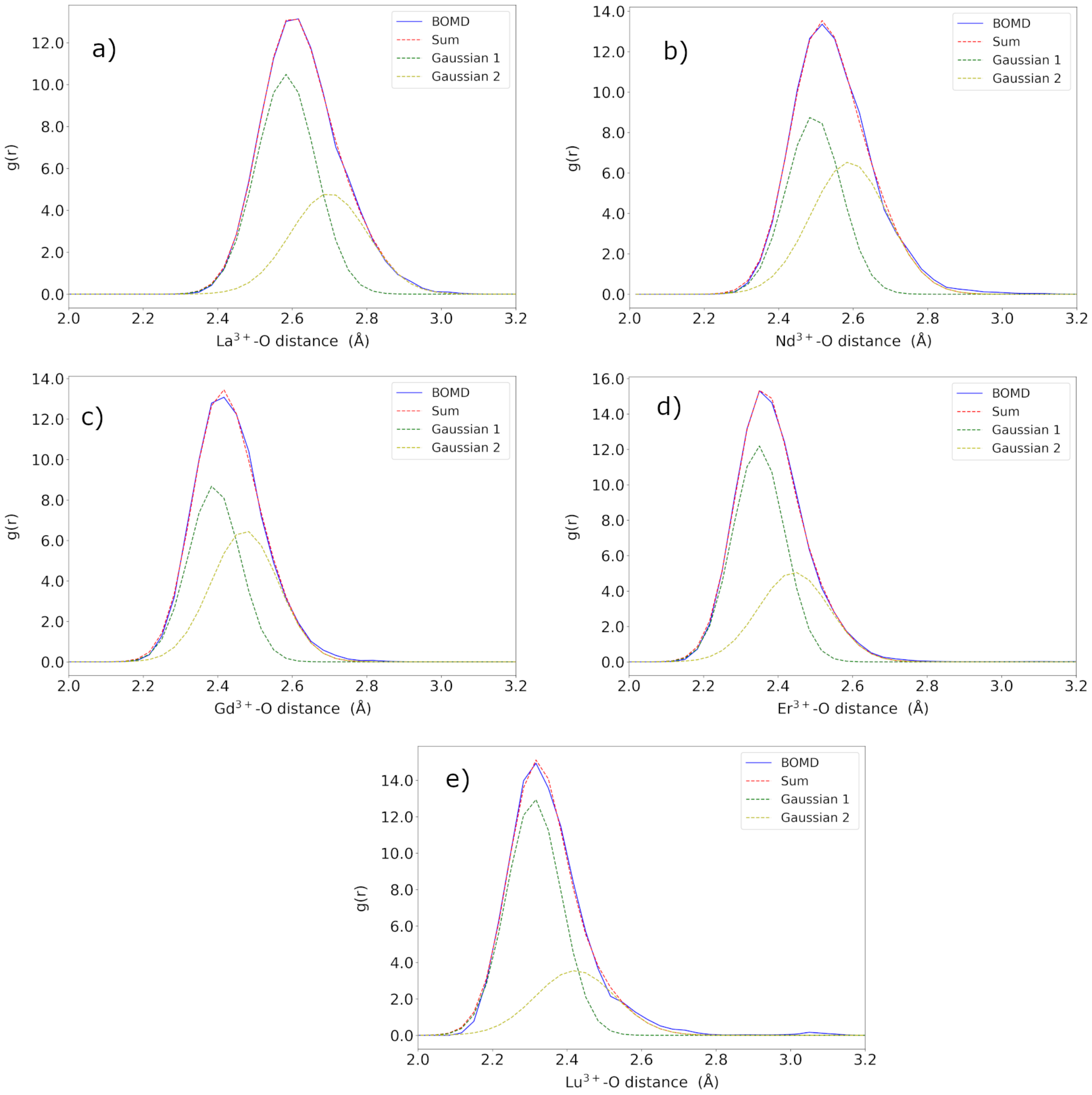


Figure S15: $Ln^{3+}$-O first peak RDFs with the fitting Gaussian curves: (a) $La^{3+}$, (b) $Nd^{3+}$, (c) $Gd^{3+}$, (d) $Er^{3+}$, and (e) $Lu^{3+}$.

# RMSDs vs. time step plots of the BOMD simulations

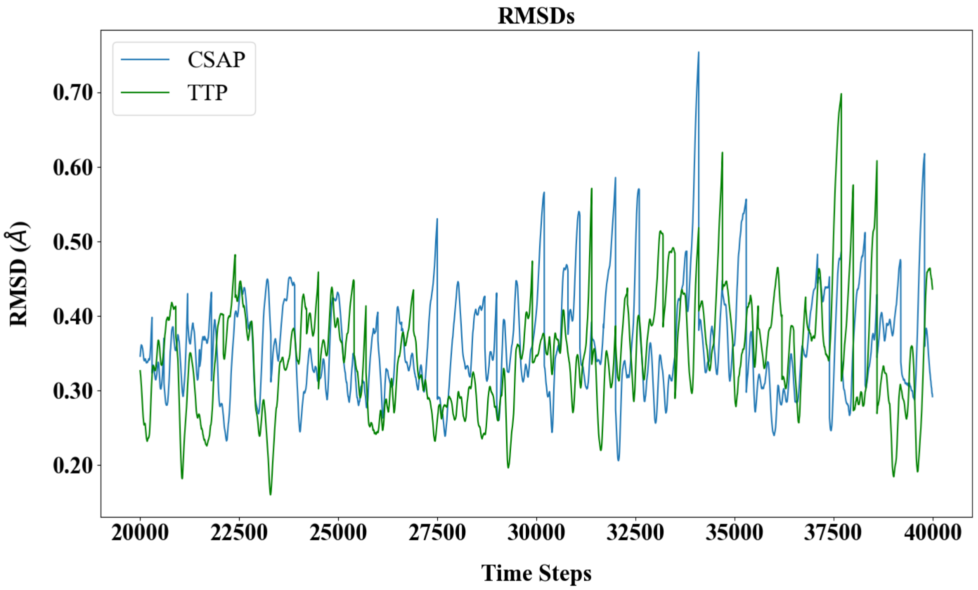


Figure S16: RMSD of the $La^{3+}$ first hydration shell with respect to reference molecular geometries along the last 10 ps of BOMD simulation.

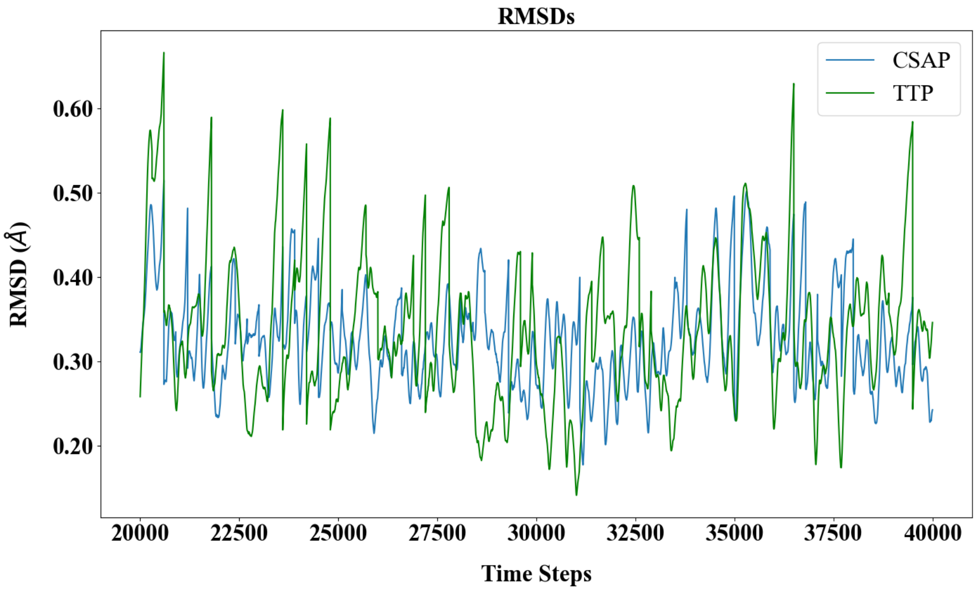


Figure S17: RMSD of the $Nd^{3+}$ first hydration shell with respect to reference molecular geometries along the the last 10 ps of BOMD simulation.

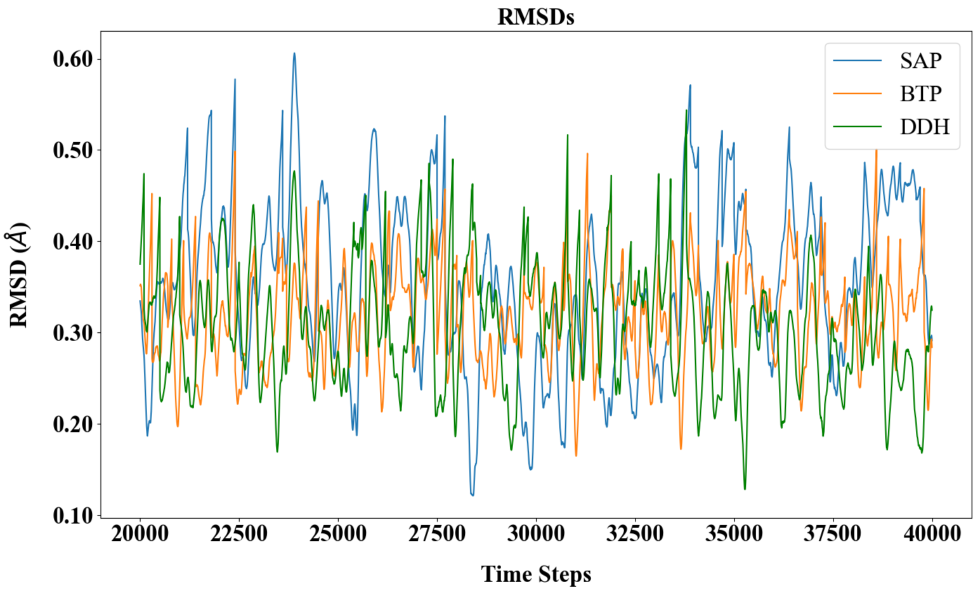


Figure S18: RMSD of the $Gd^{3+}$ first hydration shell with respect to reference molecular geometries along the last 10 ps of BOMD simulation.

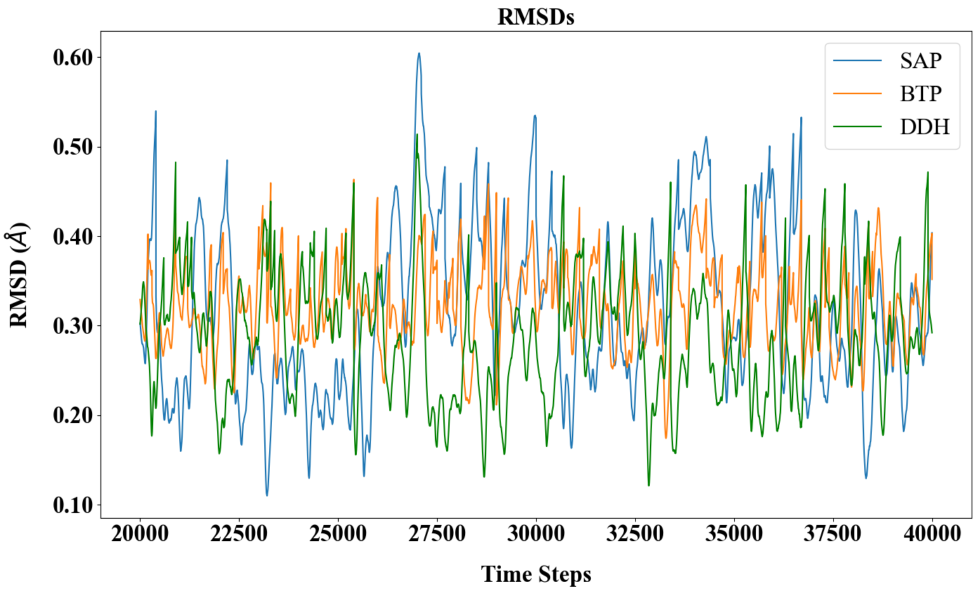


Figure S19: RMSD of the $Er^{3+}$ first hydration shell with respect to reference molecular geometries along the last 10 ps of BOMD simulation.

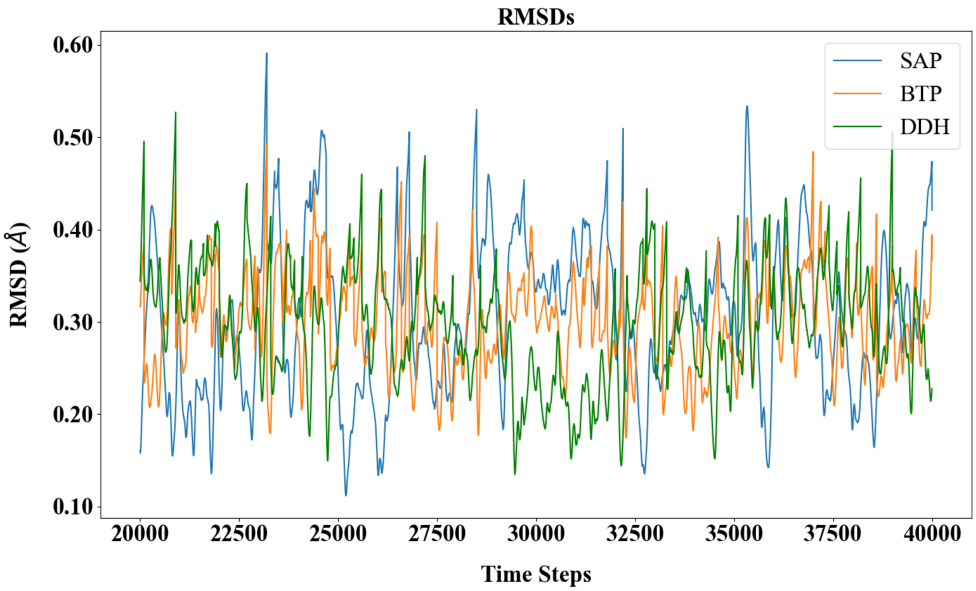


Figure S20: RMSD of the $Lu^{3+}$ first hydration shell with respect to reference molecular geometries along the last 10 ps of BOMD simulation.

# Sensitivity of the results to the number of water molecules in the complex

At first glance, the number of water molecules in this study might seem too low, as can be argued that having 16 to 18 water molecules outside the first hydration shell is insufficient to fully hydrate it. To address this concern, additional simulations were performed to support the conclusion that in periods within tens of picoseconds, the structure and dynamics of the first hydration shell of highly charged cations is affected solely by the local interactions among the cation and the water molecules in the first and second shells:

1. We produced an $Er^{3+}(H_2O)_{64}$ complex as follows: First, a 10 ns conventional molecular dynamics simulation was conducted using GROMACS 2024.1.[18–20] The initial structure was built by placing an Er ion in the center of a 32 Å wide simulation box filled with TIP3P water molecules. Energy minimization was performed with a convergence criterion of 10 kJ/mol. Next, an equilibration run in the NVT ensemble was carried

out for 200 ps using the Nose-Hoover thermostat at 300 K. This was followed by a 10 ns NPT run with the Parrinello-Rahman barostat at 1 atm. The Lennard-Jones parameters for $Er^{3+}$ are those of Qiao *et al.*[21] We then extracted the coordinates of the ion and the 64 closest water molecules. This structure was minimized at the LCECP/PBE/SV(P) level of theory using ORCA 6.0. The BOMD setup was identical to the one described in the main text. We then performed 10 ps of BOMD simulation for this $Er(H_2O)_{64}^{3+}$ complex. The system quickly equilibrated, and we used the final 9 ps to compute the relevant properties.

2. A Classical MD simulation of $Nd^{3+}$ in 139725 SPC/E water molecules within an actual nanodrop, with a radius of 10 nm, in a 40 X 40 X 40 $nm^3$ box, to avoid the use of periodic boundary conditions. The sampling was made of the NVT ensemble with the GROMACS MD-engine for a simulated time of 1 ns; the temperature was kept at 300 K with the V-rescale thermostat. The parameters for the $Nd^{3+}$ cation were those calibrated by Migliorati *et al.*[22] to reproduce the hydration free energy with the SPC/E water model.

3. A Classical MD simulation of a $Nd^{3+}$ cation with a first nonacoordinated shell, surrounded by other 55 water molecules, for a total of 64; the conditions of the simulation were the same as for the nanodrop, but the simulated time amounted to only 100 ps.

From these simulations, we computed the RDFs, performed the structural classification based on the count of equatorial oxygen atoms, and also calculated the CDFs to compare with the results presented in the main text.

## RDFs

The Er-O RDF resulting from the BOMD of the $Er(H_2O)_8(H_2O)_{56}^{3+}$ complex, labeled as Er-64, is compared to that of the 10 ps trajectory of the smaller cluster, Er-8-16, in Fig. S21. The heights of the maxima at r = 2.36 A had to be made equal for comparison. The part of

the graph that corresponds to the first hydration shells, from r = 2.0 A to r = 3.0 A, turned out slightly wider for Er-64 than for Er-8-16, but not enough to change the coordination of eight (CN's shown with discontinuous lines). The second shell starts showing at r = 3.75 A in both BOMD's. Of course, it accommodates more molecules for Er-64.

The RDF's and CN's of the simulations labeled "Classical MD nanodrop" and "Classical MD Nd-64" are depicted at the bottom rectangle of Fig. S21 and compared to those of the Nd-9-18 BOMD. The curves were rescaled to produce the same height at the first maximum at r = 2.52 A. The radial distribution of water molecules in the first shell turned out to be identical with both classical MD's and narrower than that from the Nd-9-18 BOMD. This is in agreement with the finding of Migliorati *et al.*,[22] that this slenderizing is due to the Lennard-Jones potential that yields highly structured hydration shells.

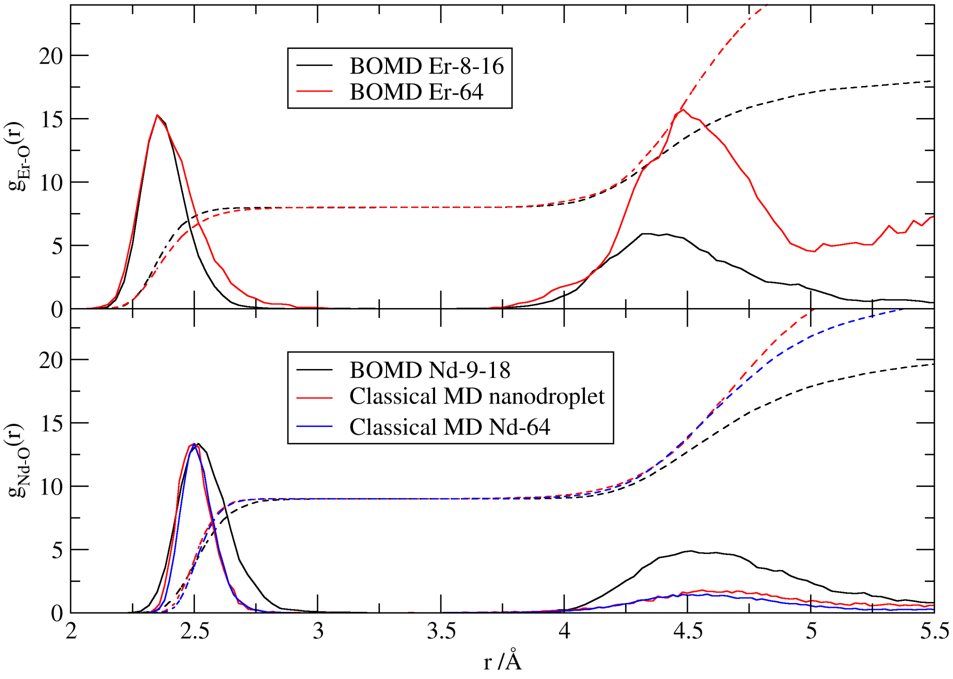


Figure S21: Comparison of the $Er^{3+}$-O and $Nd^{3+}$-O RDF's (continuous lines) and CN's (discontinuous lines) obtained from different simulations. Top: BOMD Er-8-16 from a simulation with a total of 24 water molecules, and BOMD Er-64 with 64 water molecules. Bottom: BOMD Nd-9-18, with a total of 27 water molecules; Classical MD nanodroplet, with a total of 139725 SPC/E water molecules, and Classical Nd-64, with 64 water molecules.

## Internal dynamics of the first hydration shell

The results of the classification based on the diagonalization of the inertia tensor for the polyhedron formed by the oxygens of waters belonging to the first hydration shell are presented in Tables S7 and S8 for $Nd^{3+}$ and $Er^{3+}$, respectively.

For $Nd^{3+}$, the percentage of TTPS or BTPS predicted by BOMD is consistent (within 2 units) with the values obtained from the conventional force-field-based MD approach. This indicates that our BOMD approach accurately reproduces the near-hydration structure of these ions.

For $Er^{3+}$, the results of the structural classification are nearly invariant to the number of water molecules in the simulation. Once again, this strongly suggests that our BOMD approach with 24 water molecules accurately captures the near solvation environment of these ions. The major difference lies in the results of the DDH count, for the 64 water molecule complex we obtain a 5-fold decrease in the percentage of DDHs. The major difference lies in the results for the DDH count: for the 64-water-molecule complex, we observe a 5-fold decrease in the percentage of DDHs.

Table S7: Relative frequencies of structures are presented as percentages (%) and categorized based on negative and positive Similarity Index (SI) values, as well as TTP-like and CSAP-like structures. For CSAP-like structures, the percentage (%) of cases in which the capped oxygen is situated within a 10° cone around the main inertial axis ($CSAP_{ax}$) is also provided. The last two columns display the relative frequencies (%) of TTP-like structures with SI< 0 and CSAP-like structures with SI> 0.

| | SI< 0 | SI> 0 | TTP | CSAP | $CSAP_{ax}$ | TTP(SI< 0) | CSAP(SI> 0) |
|---|---|---|---|---|---|---|---|
| $Nd(H_2O)_9(H_2O)_{18}^{3+}$ | 52.2 | 47.8 | 18.8 | 12.8 | 20.2 | 45.0 | 30.0 |
| Nd-nanodrop | 52.0 | 48.0 | 21.1 | 13.0 | 33.1 | 40.3 | 45.4 |

Table S8: Relative frequencies of structures are presented as percentages (%) and categorized based on negative and positive Similarity Index (SI) values, as well as BTP-like and SAP-like. The two following columns display the relative frequencies (%) of BTP-like structures with SI< 0 and SAP-like structures with SI> 0. The last column has the relative frequencies of putative DDH-like structures found by applying the geometrical criterion of two perpendicular planes, each with four oxygens and both comprising the lanthanoid.[23]

| | SI< 0 | SI> 0 | BTP | SAP | BTP(SI< 0) | SAP(SI> 0 ) | DDH |
|---|---|---|---|---|---|---|---|
| $Nd(H_2O)_8(H_2O)_{16}^{3+}$ | 60.2 | 39.8 | 21.9 | 43.1 | 88.5 | 73.1 | 8.3 |
| $Nd(H_2O)_8(H_2O)_{54}^{3+}$ | 58.7 | 41.3 | 25.4 | 41.4 | 84.9 | 70.3 | 1.6 |

## CDFs

The accurate modeling of the near-hydration structure within our BOMD approach is further supported by the CDFs results shown in Figures S22 and S23.

For $Nd^{3+}$, the CDFs exhibit the same radial-angular distribution regardless of the method used. It is worth noting the narrower width of the CDF obtained with conventional force-field-based MD is expected, as discussed earlier. Nevertheless, both CDFs highlight the fluxional nature of the first hydration shell.

In the case of $Er^{3+}$, the CDF with 64 water molecules shows slightly broader peaks compared to the one with 24 water molecules. However, the overall picture remains unchanged: the radial-angular distribution is highly dynamic, preventing the determination of a dominant binding conformation

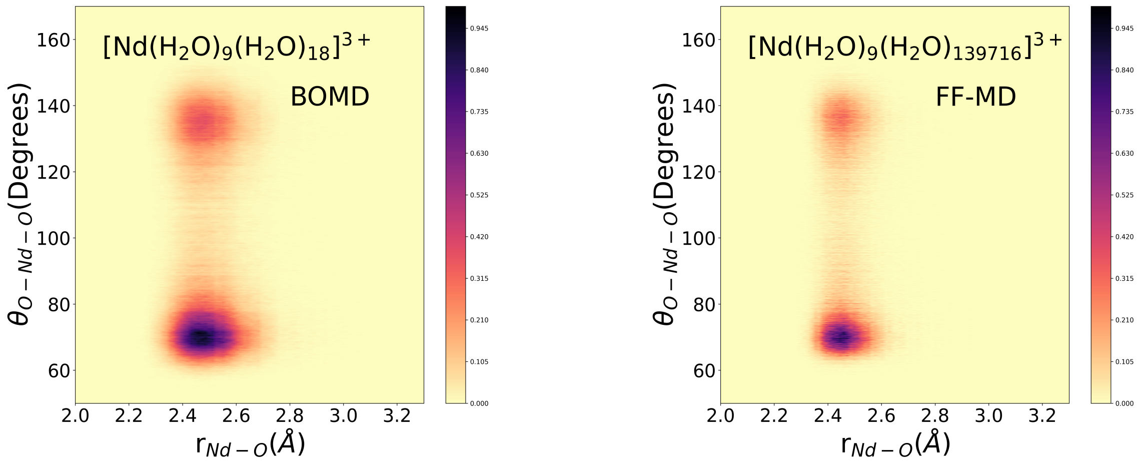


(a) CDF evaluated from the BOMD of the $Nd(H_2O)_9(H_2O)_{18}^{3+}$ complex.

(b) CDF evaluated from the FF-MD of the $Nd(H_2O)_9(H_2O)_{139716}^{3+}$ complex.

Figure S22: Comparison of the computed CDFs for nanodroplets containing 24 (BOMD) vs. 139725 (FF-MD) water molecules.

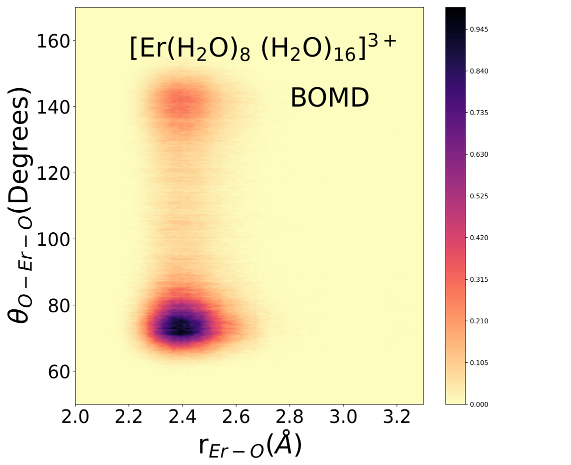


(a) CDF evaluated from the BOMD of the $Er(H_2O)_8(H_2O)_{16}^{3+}$ complex.

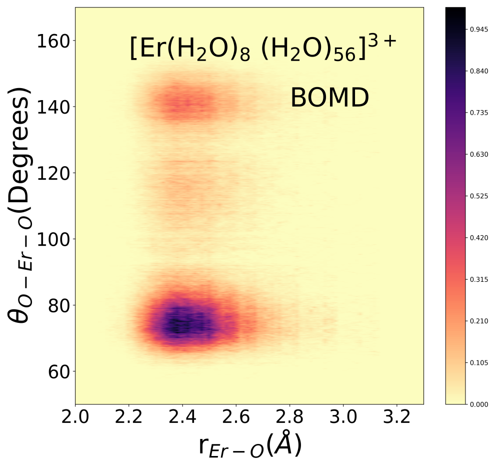


(b) CDF evaluated from the BOMD of the $Er(H_2O)_8(H_2O)_{64}^{3+}$ complex.

Figure S23: Comparison of the computed CDFs for nanodroplets containing 24 vs. 64 water molecules.

# References


(1) Ciupka, J.; Cao-Dolg, X.; Wiebke, J.; Dolg, M. Computational study of lanthanide (III) hydration. *Physical Chemistry Chemical Physics* **2010**, *12*, 13215–13223.

(2) Bryantsev, V. S.; Diallo, M. S.; Goddard Iii, W. A. Calculation of solvation free energies of charged solutes using mixed cluster/continuum models. *The Journal of Physical Chemistry B* **2008**, *112*, 9709–9719.

(3) Kuta, J.; Clark, A. E. Trends in aqueous hydration across the 4f period assessed by reliable computational methods. *Inorganic chemistry* **2010**, *49*, 7808–7817.

(4) Zhang, J.; Heinz, N.; Dolg, M. Understanding lanthanoid (III) hydration structure and kinetics by insights from energies and wave functions. *Inorganic Chemistry* **2014**, *53*, 7700–7708.

(5) Clark, A. E. Density functional and basis set dependence of hydrated Ln (III) properties. *Journal of Chemical Theory and Computation* **2008**, *4*, 708–718.

(6) Habenschuss, A.; Spedding, F. H. The coordination (hydration) of rare earth ions in aqueous chloride solutions from x ray diffraction. I.TbCl3,DyCl3, ErCl3,TmCl3,and LuCl3. *The Journal of Chemical Physics* **2008**, *70*, 2797–2806.

(7) Habenschuss, A.; Spedding, F. H. The coordination (hydration) of rare earth ions in aqueous chloride solutions from x-ray diffraction. II. LaCl3, PrCl3, and NdCl3a). *The Journal of Chemical Physics* **2008**, *70*, 3758–3763.

(8) Habenschuss, A.; Spedding, F. H. The coordination (hydration) of rare earth ions in aqueous chloride solutions from x-ray diffraction. III. $SmCl_3$, $EuCl_3$, and series behavior. *The Journal of Chemical Physics* **1980**, *73*, 442–450.

(9) Yamaguchi, T.; Nomura, M.; Wakita, H.; Ohtaki, H. An extended x-ray absorption fine structure study of aqueous rare earth perchlorate solutions in liquid and glassy states. *The Journal of chemical physics* **1988**, *89*, 5153–5159.

(10) Allen, P.; Bucher, J.; Shuh, D.; Edelstein, N.; Craig, I. Coordination chemistry of trivalent lanthanide and actinide ions in dilute and concentrated chloride solutions. *Inorganic Chemistry* **2000**, *39*, 595–601.

(11) Persson, I.; D'Angelo, P.; De Panfilis, S.; Sandström, M.; Eriksson, L. Hydration of lanthanoid (III) ions in aqueous solution and crystalline hydrates studied by EXAFS spectroscopy and crystallography: The myth of the "gadolinium break". *Chemistry–A European Journal* **2008**, *14*, 3056–3066.

(12) D'Angelo, P.; Zitolo, A.; Migliorati, V.; Chillemi, G.; Duvail, M.; Vitorge, P.; Abadie, S.; Spezia, R. Revised ionic radii of lanthanoid (III) ions in aqueous solution. *Inorganic chemistry* **2011**, *50*, 4572–4579.

(13) Marcus, Y. A simple empirical model describing the thermodynamics of hydration of ions of widely varying charges, sizes, and shapes. *Biophysical chemistry* **1994**, *51*, 111–127.

(14) Shiery, R. C.; Fulton, J. L.; Balasubramanian, M.; Nguyen, M.-T.; Lu, J.-B.; Li, J.; Rousseau, R.; Glezakou, V.-A.; Cantu, D. C. Coordination sphere of lanthanide aqua ions resolved with ab initio molecular dynamics and X-ray absorption spectroscopy. *Inorganic Chemistry* **2021**, *60*, 3117–3130.

(15) McElhany, S. J.; Summers, T. J.; Shiery, R. C.; Cantu, D. C. Analysis of the First Ion Coordination Sphere: A Toolkit to Analyze the Coordination Sphere of Ions. *Journal of Chemical Information and Modeling* **2023**, *63*, 2699–2706.

(16) Helm, L.; Merbach, A. Structure and dynamics of lanthanide (III) ions in solution: a neutron scattering contribution. *European Journal of Solid State and Inorganic Chemistry;(France)* **1991**, *28*.

(17) Kowall, T.; Foglia, F.; Helm, L.; Merbach, A. Molecular dynamics simulation study of lanthanide ions Ln3+ in aqueous solution. Analysis of the structure of the first hydration shell and of the origin of symmetry fluctuations. *The Journal of Physical Chemistry* **1995**, *99*, 13078–13087.

(18) Van Der Spoel, D.; Lindahl, E.; Hess, B.; Groenhof, G.; Mark, A. E.; Berendsen, H. J. GROMACS: fast, flexible, and free. *Journal of computational chemistry* **2005**, *26*, 1701–1718.

(19) Abraham, M. J.; Murtola, T.; Schulz, R.; Páll, S.; Smith, J. C.; Hess, B.; Lindahl, E. GROMACS: High performance molecular simulations through multi-level parallelism from laptops to supercomputers. *SoftwareX* **2015**, *1*, 19–25.

(20) Abraham, M. J.; et al. GROMACS 2024.1 Manual. version 2024.1, 2024.

(21) Qiao, B.; Skanthakumar, S.; Soderholm, L. Comparative CHARMM and AMOEBA simulations of lanthanide hydration energetics and experimental aqueous-solution structures. *Journal of chemical theory and computation* **2018**, *14*, 1781–1790.

(22) Migliorati, V.; Serva, A.; Terenzio, F. M.; D'Angelo, P. Development of Lennard-Jones and Buckingham potentials for lanthanoid ions in water. *Inorganic chemistry* **2017**, *56*, 6214–6224.

(23) Drew, M. G. Structures of high coordination complexes. *Coordination Chemistry Reviews* **1977**, *24*, 179–275.
